# Quadrupolar Dyes Based on Highly Polarized Coumarins

**DOI:** 10.1021/acs.orglett.1c02349

**Published:** 2021-08-16

**Authors:** Krzysztof Górski, Irena Deperasińska, Glib V. Baryshnikov, Shuhei Ozaki, Kenji Kamada, Hans Ågren, Daniel T. Gryko

**Affiliations:** † Institute of Organic Chemistry, Polish Academy of Sciences, Kasprzaka 44/52, 01-224 Warsaw, Poland; ‡ Institute of Physics, Polish Academy of Sciences, Al. Lotników 32/46, 02-668 Warsaw, Poland; § Department of Physics and Astronomy, Uppsala University, Box 516, SE-751 20 Uppsala, Sweden; ∥ Nanomaterials Research Institute (NMRI), National Institute of Advanced Industrial Science and Technology (AIST), Ikeda, Osaka 563-8577, Japan; ⊥ Department of Chemistry, Graduate School of Science and Technology, Kwansei Gakuin University, Sanda 669-1337, Japan; # Laboratory of Organic Electronics, Department of Science and Technology, Linköping University, SE-60174 Norrköping, Sweden

## Abstract

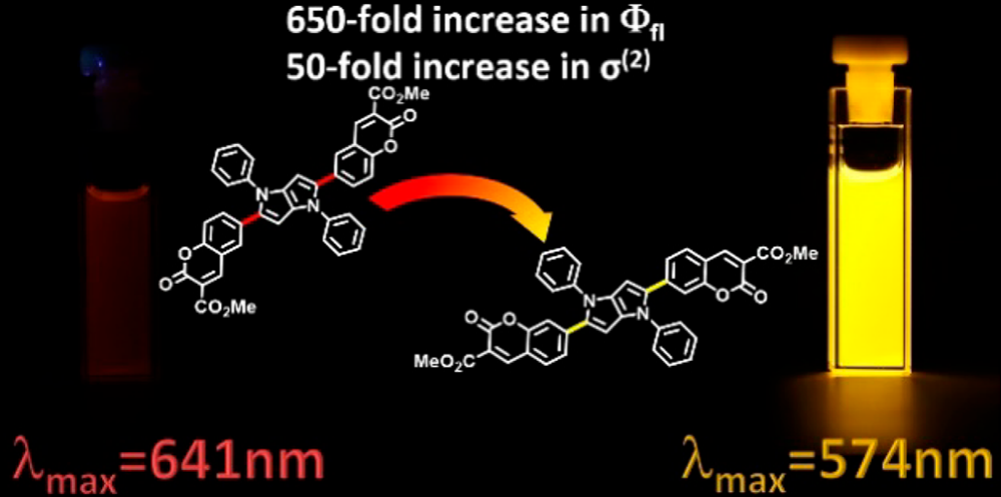

The fluorescence and other photophysical parameters of highly polarized, quadrupolar bis-coumarins possessing an electron-rich pyrrolo[3,2-*b*]pyrrole bridging unit are highly dependent on the linking position between both chromophores. Delocalization of the LUMO on the entire π-system results in intense emission and strong two-photon absorption.

Coumarins are a large family of oxygen-containing heterocycles which were first isolated 200 years ago. Since then, interest in this class of substances has remained strong owing to the many derivatives that display broad biological activity.^1,2^ Coumarins have also attracted significant scientific attention due to their unique photophysical properties.^3−5^ Their simple synthesis combined with the relative ease of functionalization makes it possible to create a wide range of dyes.^6^ A particularly important class of coumarin-based emitters are donor–acceptor systems possessing an electron-donating substituent at position 7 and an electron-withdrawing one at position 3.^7,8^ It is well-known that 7-aminocoumarins exhibit excellent emissive properties.^9^ Intriguingly, switching the position of an electron-donating substituent from 7 to 6 leads to marked differences in optoelectronic properties.^10−14^ In the case of 6-aminocoumarins the emission is weak and red-shifted in comparison with the corresponding 7-aminocoumarins.^15,16^ In this letter we address a key question: is this a general effect of electron-donating substituents irrespective of their structure? To answer this query we adopted a pyrrolo[3,2-*b*]pyrrole (PP) scaffold as the bridge between two coumarins. It was chosen because of its exceptional electron-donating character. At the same time, the newly designed dyes constitute the first quadrupolar, centrosymmetric, acceptor–donor–acceptor (A–D–A) architecture that possesses coumarin units, which can be an excellent two-photon absorber.^13,17^

Numerous studies have shown that in pyrrolo[3,2-*b*]pyrroles the electronic communication is particularly strong at positions 2 and 5.^18^ In order to install coumarin units into these locations on this heterocycle, formyl-coumarin derivatives were designed for the recently optimized multicomponent reaction between aromatic amines, butane-2,3-dione, and aromatic aldehydes for the pyrrolo[3,2-*b*]pyrrole synthesis.^19^ The formyl-coumarin derivatives were additionally designed to possess an auxiliary CO_2_Me group at position 3 in order to increase their accepting character. The isomeric 6- and 7-formyl coumarins **2a** and **2b** designed for this purpose are shown in Scheme 1.

**Scheme 1:**
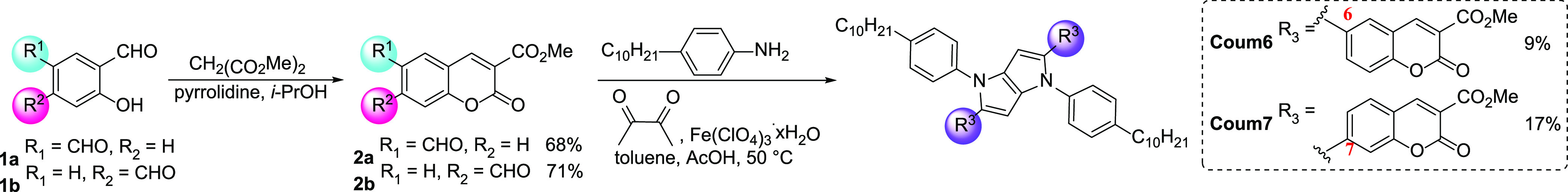
Synthesis of Bis(coumarin)pyrrolopyrroles **Coum6** and **Coum7**

The first attempts at performing the Knoevenagel condensation between dialdehyde **1a** and dimethyl malonate in methanol did not give the desired product **2a**. Due to the presence of two aldehyde groups in compound **1a**, a significant portion of the isolated material was the double condensation product. Subsequent experiments finally made it possible to obtain **2a** in satisfactory yield (68%) through the use of isopropanol as the reaction medium in which the solubility of **2a** is significantly lower than in methanol, leading to the precipitation of the desired product from the reaction mixture.

The last step in the synthetic pathway leading to regioisomeric A–D–A systems was the multicomponent condensation between **2a** and **2b**, 4-decylaniline, and butane-2,3-dione in the presence of a catalytic amount of iron(III) perchlorate. Thus, **Coum6** with a yield of 9%, and **Coum7** with a yield of 17% were obtained (Scheme 1). The 2-fold higher yield of **Coum7** is probably the result of the orientation of the formyl group in the aldehyde **2b** being in the *para* position to the malonylidene subunit, which increases the electron deficiency within the formyl group, enhancing its reactivity toward the nucleophiles. To interrogate the role of the bridging position on the photophysical properties, absorption and emission measurements were carried out in three solvents differing in polarity (Figures 1 and 2, Table 1). The differences in the spectroscopic properties of isomeric A–D–A dyes become visible by the naked eye (**Coum6** - orange solid, **Coum7** - red solid). **Coum6** exhibits intense UV light absorption (λ_abs_ = 358 nm), whereas in the low-energy part of the absorption spectrum a weak, broad band can be observed (λ_abs_ = 440 nm). The opposite effect occurs in the case of **Coum7**, where strong absorption of yellow light (λ_abs_ = 485 nm) is accompanied by a residual absorption of UV radiation. In accordance with the centrosymmetric architecture, there is no solvatochromism in these dyes; however, a significant drop of absorption coefficient is observed while the polarity of the solvent increases (Figures 1 and 2).

**Figure 1:**
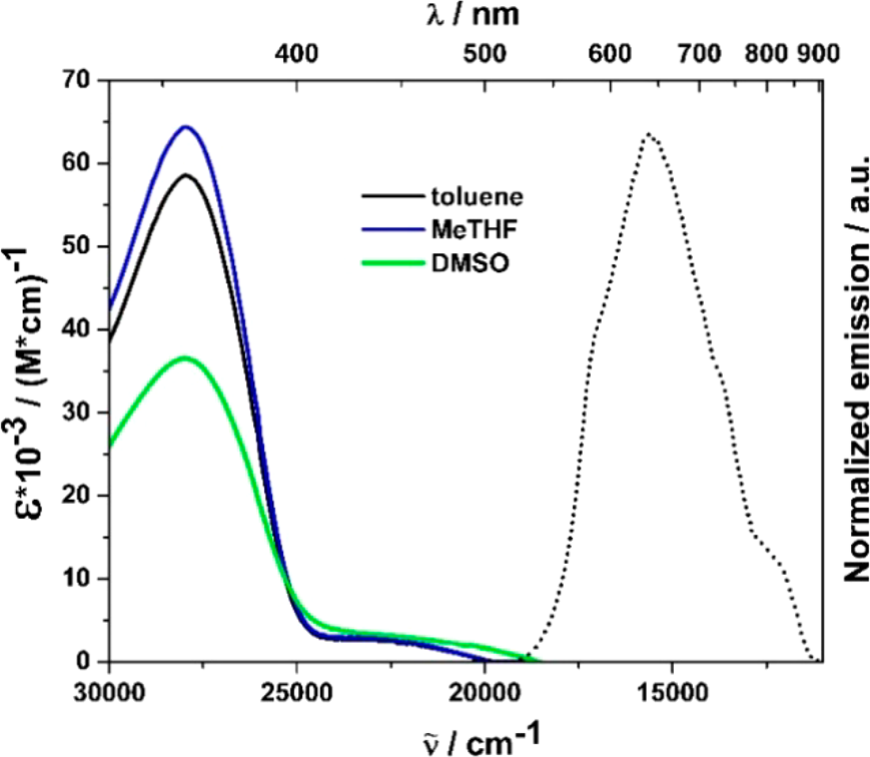
Absorption (solid line) and normalized fluorescence (dotted line) spectra of **Coum6** measured in three different solvents recorded with excitation at 354 nm. Legend specifies colors of lines.

**Figure 2:**
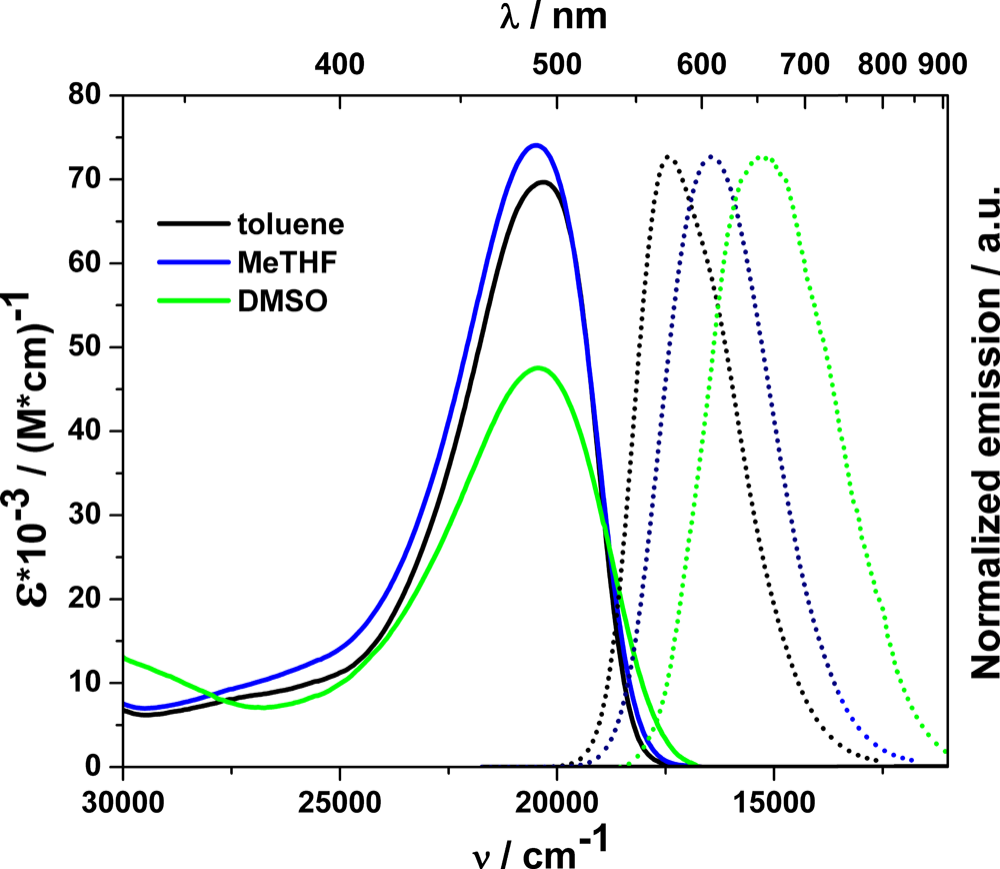
Absorption (solid line) and normalized fluorescence (dotted line) spectra of **Coum7** measured in three different solvents recorded with excitation at 440 nm. Legend specifies colors of lines.

**Table 1:** Photophysical Data of **Coum6** and **Coum7** Measured in Solution

Dye	Solvent	λ_abs_^max^ [nm]	λ_em_^max^ [nm]	Φ_fl_ [%]	*Δν* [cm^–1^]
**Coum6**	toluene	358, 439	641	0.06^a^	7200
	MeTHF	358, 439	–	–	–
	DMSO	358, 440	–	–	–
**Coum7**	toluene	486	574	39^b^	3000
	MeTHF	485	608	30^b^	4100
	DMSO	487	649	0.02^b^	5300

a Reference: 9,10-diphenylantracene in toluene (Φ_fl_ = 0.70).

b Reference: Coumarin 153 in toluene (Φ_fl_ = 0.40).

There are two strong analogies between the above-described characteristics and photophysics of simpler D–A coumarins. First, in **Coum6** the Stokes shift is large whereas there is only a moderate difference between the absorption and emission in the case of **Coum7**. Moreover, for **Coum6** a very weak red fluorescence is observed, while **Coum7** has strong emission (Table 1). It should also be mentioned that, for more polar solvents, the **Coum6** emission is below the detection limit. On the other hand, changing the substitution position on the coumarin subunit from 6 to 7 results in a 650-fold increase in the fluorescence quantum yield from 0.06% to 39% in toluene. Due to the incomparably stronger emissive properties, in the case of **Coum7**, solvatofluorochromism can be observed indicating excited-state symmetry-breaking.^20^ The successive increase in the solvent polarity results in a clear Stokes shift, from 3000 cm^–1^ in toluene to 5300 cm^–1^ in DMSO. The described phenomenon is also accompanied by a significant decrease of the fluorescence quantum yield, down to 0.02% in DMSO.

In principle the photophysical properties of these quadrupolar bis-coumarins mirror the properties of 7-aminocoumarins vs 6-aminocoumarins; i.e., coumarins possessing electron-donating groups at the 6-position have weak but bathochromically shifted emission whereas coumarins substituted at the 7-position exhibit strong emission.

From a purely structural perspective the investigated dyes can be considered as bis-coumarins and, at the same time, as centrosymmetric pyrrolo[3,2-*b*]pyrroles. The photophysical properties of **Coum6** and **Coum7** can be directly compared to bis-2,5-(4-cyanophenyl)pyrrolo[3,2-*b*]pyrrole,^18^ which is the prototypical A–D–A pyrrolo[3,2-*b*]pyrrole. Absorption of **Coum7** is bathochromically shifted by ca. 80 nm and the emission by over 120 nm, which reveals that the coumarin scaffolds affect the electron structure making it a truly π-expanded system. On the other hand, the main absorption band of **Coum6** is hypsochromically shifted ca. 50 nm in comparison to bis-2,5-(4-cyanophenyl)pyrrolo[3,2-*b*]pyrrole.

The investigated dyes A–D–A architecture encouraged us to explore their two-photon absorption (TPA) properties, as A–D–A is one of the most successful motifs of TPA dyes. The TPA spectra were measured using a femtosecond open-aperture Z-scan method^21,22^ in a MeTHF (Figure 3). **Coum7** was found to have a strong and broad TPA peak centered at 12 000 cm^–1^ (840 nm) with the peak TPA cross section equal to 850 ± 160 GM, where 1 GM = 10^–50^ cm^4^ s photon^–1^ molecule^–1^. The two-photon absorption cross section increased even further starting from 650 nm toward the blue edge of the spectrum, while the σ^(2)^ reached 4000 ± 430 GM at 17 500 cm^–1^ (453 nm). In contrast, **Coum6** showed a much weaker TPA, with σ^(2)^*s* ≤ 10 GM for 9500–15000 cm^–1^ (1050–660 nm). The spectral magnitude was monotonically increased at the photon energy higher than 15 000 cm^–1^ (Figure S17); nevertheless, the maximum value observed was σ^(2)^ = 100 ± 26 GM at 17 500 cm^–1^ (453 nm), which is in 40-fold contrast to that of **Coum7**.

**Figure 3:**
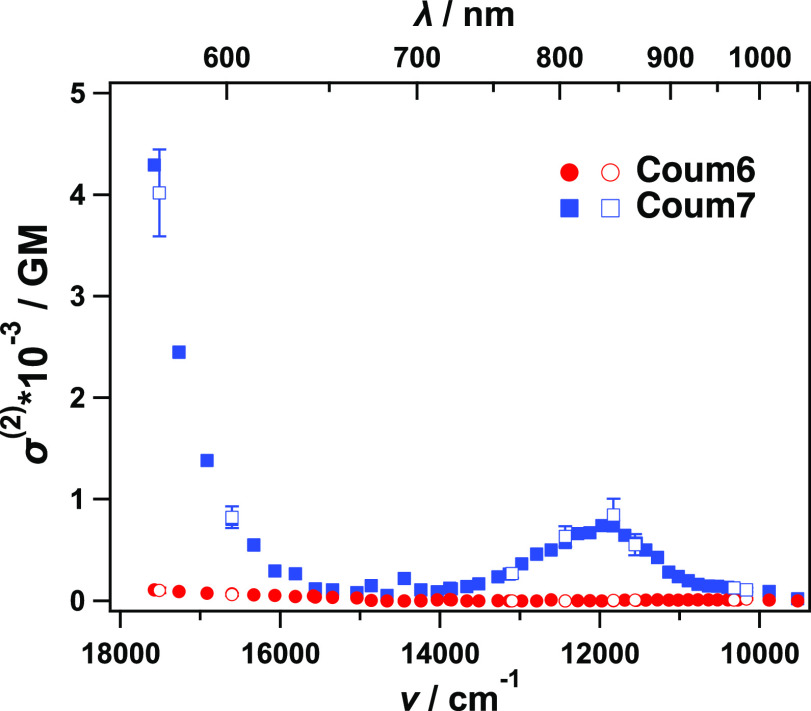
Two-photon absorption spectra of **Coum6** (red circles) and **Coum7** (blue squares) both in MeTHF. Solid symbols represent data measured at fixed excitation powers. Contoured symbols with error bars show data obtained through confirming its excitation intensity dependence.

The TPA peak of **Coum7** at 12 000 cm^–1^ (i.e., 24 000 cm^–1^ in the transition energy) did not match the transition energy produced by the one-photon absorption peak (Figure 2). This behavior can be understood using Laporte’s selection rule complementary for one- and two-photon absorption. The significant increase observed at higher energies likely originates from a resonance enhancement.^23^

In order to investigate the effect of the coumarin substitution site on the photophysical properties, we combined our experimental results with TD-DFT calculations (including optimization of structures) using different functionals and basis sets (see Supporting Information (SI) for details). We note that the use of the standard TD DFT/B3LYP/6-31G(d,p) approach in the case of CT systems like **Coum6** and **Coum7** is insufficient to properly reproduce the photophysical properties. To obtain better agreement between experiment and computational results, hybrid functionals with an increased amount of Hartree–Fock exchange, such as B3LYP-37, should be used. Calculated energies of electronic transitions with that functional are shown in Table 2. Small differences between experiment and calculations result from the limitations of the TD-DFT method and from the fact that molecules used in calculations do not possess alkyl chains at phenyl and carboxyl subunits.

**Table 2:** PCM/B3LYP-37/6-31G(d,p) Calculation Results of **Coum6** and **Coum7** Electronic Transitions in Toluene^a^

Transition	**Coum6** λ [nm] (*f*)	**Coum7** λ [nm] (*f*)
S_0_→S_1_	448 (0.062)	475 (2.089)
S_0_→S_2_	448 (0.000)	397 (0.000)
S_0_→S_3_	342 (1.914)	354 (0.051)
S_0_→S_4_	338 (0.000)	319 (0.000)
S_0_→S_5_	338 (0.055)	315 (0.000)
S_1_→S_0_	598 (0.017)	535 (2.441)

a Wavelengths (λ) and oscillator strengths (f) of the S_0_ → S_i_ electronic transitions.

As can be seen in the table, the oscillator strength of the S_0_ → S_1_ transition in **Coum6** is much lower (*f* = 0.062) compared to the S_0_ → S_1_ transition in **Coum7** (*f* = 2.089) which indicates the effectively forbidden character of the **Coum6** first electron transition, leading to a lack of absorption in the yellow region. On the other hand, the **Coum7** absorption spectra are bathochromically shifted compared to **Coum6**, which is in line with the computational results. Moreover, the observed strong absorption in the UV range for **Coum6** corresponds to the allowed S_0_ → S_3_ transition (*f* = 1.914). Due to the fact that the S_0_ → S_1_ as well as the S_1_ → S_0_ transitions are mainly described by HOMO/LUMO configurations, the two transitions possess similar properties; thus the forbidden nature of the S_0_ → S_1_ transition also manifests in the emissive properties of **Coum6**. However, an analysis of fluorescence spectra of investigated A–D–A systems indicates that, in the case of **Coum6**, a much larger Stokes shift is observed, compared to **Coum7**.

HOMOs of both **Coum6** and **Coum7** are mainly located on the electron-rich pyrrolo[3,2-*b*]pyrrole core (Figure 4). On the other hand, clear differences of the electron density can be observed for LUMOs. In the case of **Coum6**, the LUMO is completely located on the electron-accepting coumarin subunits, while the **Coum7** LUMO orbital is also localized on the central pyrrolopyrrole core. Such shapes of the frontier orbitals indicate that the S_0_ → S_1_ transitions in **Coum6** and **Coum7** are intramolecular charge-transfer (CT) transitions from D to the two A centers, with a lesser degree of charge transfer in **Coum7** (see SI for quantitative data). Moreover, upon excitation of **Coum6**, a better charge separation is observed (see SI for quantitative data). The larger charge separation in the **Coum6** S_1_ excited state leads to significant Coulomb interaction driven stabilization, manifested by the lower energy of the S_1_ → S_0_ transition and a significant drop in oscillator strength (*f* = 0.062) with regard to **Coum7** (*f* = 2.089). On the other hand, a much weaker charge separation in the **Coum7** S_1_ state, caused by the significant delocalization of the LUMO over both the pyrrolo[3,2-*b*]pyrrole core and the coumarin subunits, leads to a greater oscillator strength of the S_1_ → S_0_ transition.

**Figure 4:**
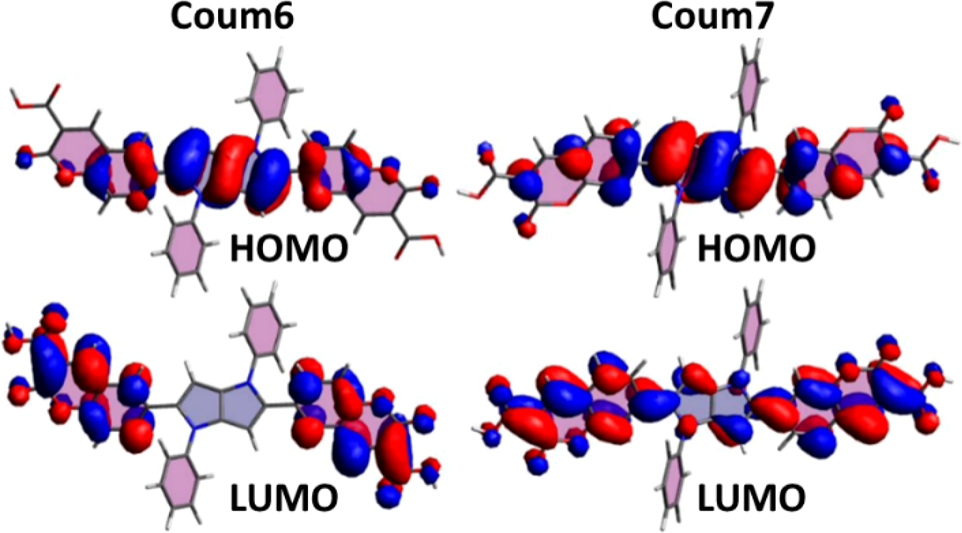
HOMO and LUMO orbitals of **Coum6** and **Coum7**.

A simulation of the TPA spectrum successfully reproduced the contrast features of **Coum6** and **Coum7** (Figure S5). For **Coum6** the transition intensity is weak for transitions up to S_8_, with strong TPA transitions only existing for those to higher excited states. In contrast, for **Coum7**, a strong transitions to S_2_ appeared at 770 nm, which corresponds to the experimentally observed TPA peak centered at 840 nm (Figure 3), though it is energetically overestimated. Calculation results also show complementary behavior of one- and two-photon transitions for these centrosymmetric molecules. Weak or no TPA transitions were observed to the excited states to which one-photon transitions are strong (S_0_ → S_3_ for **Coum6** and S_0_ → S_1_ for **Coum7**) as well as vice versa (S_0_ → S_2_ of **Coum7** is a strong TPA transition but forbidden for one-photon absorption).

By applying the concept of electron donor–acceptor systems,^24^ the differences between the transition energies and oscillator strengths for the excitation and fluorescence of **Coum6** and **Coum7** can be assigned to the differentiation of short-range interactions within the AD junction. This is in line with the results of Liu and co-workers for 6-aminocoumarins.^25^ The calculated solvent shifts of the transition energies, along with the increase of solvent polarity, are in good agreement with those observed (see SI). There is however no clear explanation for the observed decrease of fluorescence yield of **Coum7** in polar DMSO. This suggests an opening of the nonradiative decay pathway and requires the use of increasingly sophisticated models of solvent effects for CT systems.^26−28^

Both the quadrupolar architecture of the hybrid dyes and the bridging position of the coumarin scaffolds with the electron-rich pyrrolo[3,2-*b*]pyrrole unit play decisive roles in the optoelectronic properties of the new dyes. In analogy to the classical 7-dialkylaminocoumarins, the emission of the quadrupolar bis-coumarin with a pyrrolo[3,2-*b*]pyrrole unit at the seventh position is strong and moderately bathochromically shifted. Shifting the bridge to the position 6 drastically changes the nature of the LUMO, resulting in its localization solely on the coumarin subunits. This results in a weakly emitting dye with λ_em_^max^ at 650 nm. Marked differences in their two-photon absorbing properties (TPA cross-section in the near-infrared region decreased from 850 GM for **Coum7** to less than 10 GM for **Coum6**) are also caused by the diversity of the LUMO distribution.

## Supporting Information Available

Quantum chemical calculation and photophysical experimental data, and synthetic procedures, as well as ^1^H and ^13^C{^1^H} NMR spectra (PDF)

## References

[ref1] Stefanachi A.; Leonetti F.; Pisani L.; Catto M.; Carotti A. Coumarin: A Natural, Privileged and Versatile Scaffold for Bioactive Compounds. Molecules 2018, 23 (2), 250 10.3390/molecules23020250. PMC6017103 29382051

[ref2] Srikrishna D.; Godugu C.; Dubey P. K. A Review on Pharmacological Properties of Coumarins. Mini-Rev. Med. Chem. 2018, 18 (2), 113–141. 10.2174/1389557516666160801094919. 27488585

[ref3] Tsukamoto K.; Shinohara Y.; Iwasaki S.; Maeda H. A Coumarin-Based Fluorescent Probe for Hg2+ and Ag+ with an N′-Acetylthioureido Group as a Fluorescence Switch. Chem. Commun. 2011, 47 (17), 5073–5075. 10.1039/c1cc10933b. 21431242

[ref4] Adronov A.; Gilat S. L.; Fréchet J. M. J.; Ohta K.; Neuwahl F. V. R.; Fleming G. R. Light Harvesting and Energy Transfer in Laser–Dye-Labeled Poly(Aryl Ether) Dendrimers. J. Am. Chem. Soc. 2000, 122 (6), 1175–1185. 10.1021/ja993272e.

[ref5] Liu X.; Xu Z.; Cole J. M. Molecular Design of UV–Vis Absorption and Emission Properties in Organic Fluorophores: Toward Larger Bathochromic Shifts, Enhanced Molar Extinction Coefficients, and Greater Stokes Shifts. J. Phys. Chem. C 2013, 117 (32), 16584–16595. 10.1021/jp404170w.

[ref6] Bassolino G.; Nançoz C.; Thiel Z.; Bois E.; Vauthey E.; Rivera-Fuentes P. Photolabile Coumarins with Improved Efficiency through Azetidinyl Substitution. Chem. Sci. 2018, 9 (2), 387–391. 10.1039/C7SC03627B. 29629108 PMC5868312

[ref7] Jones G.; Rahman M. A. Fluorescence Properties of Coumarin Laser Dyes in Aqueous Polymer Media. Chromophore Isolation in Poly(Methacrylic Acid) Hypercoils. J. Phys. Chem. 1994, 98 (49), 13028–13037. 10.1021/j100100a035.

[ref8] Kielesiński Ł.; Morawski O.; Dobrzycki Ł.; Sobolewski A. L.; Gryko D. T. The Coumarin-Dimer Spring-The Struggle between Charge Transfer and Steric Interactions. Chem. - Eur. J. 2017, 23 (38), 9174–9184. 10.1002/chem.201701387. 28500858

[ref9] Grandberg I. I.; Denisov L. K.; Popova O. A. 7-Aminocoumarins (Review). Chem. Heterocycl. Compd. 1987, 23 (2), 117–142. 10.1007/BF00663848.

[ref10] Samanta A.; Fessenden R. W. Excited-State Dipole Moment of 7-Aminocoumarins as Determined from Time-Resolved Microwave Dielectric Absorption Measurements. J. Phys. Chem. A 2000, 104 (37), 8577–8582. 10.1021/jp001676j.

[ref11] Krystkowiak E.; Dobek K.; Maciejewski A. An Intermolecular Hydrogen-Bonding Effect on Spectral and Photophysical Properties of 6-Aminocoumarin in Protic Solvents. Photochem. Photobiol. Sci. 2013, 12 (3), 446–455. 10.1039/C2PP25288K. 23178802

[ref12] Lin Q.; Bao C.; Fan G.; Cheng S.; Liu H.; Liu Z.; Zhu L. 7-Amino Coumarin Based Fluorescent Phototriggers Coupled with Nano/Bio-Conjugated Bonds: Synthesis, Labeling and Photorelease. J. Mater. Chem. 2012, 22 (14), 6680–6688. 10.1039/c2jm30357d.

[ref13] Klausen M.; Dubois V.; Clermont G.; Tonnelé C.; Castet F.; Blanchard-Desce M. Dual-Wavelength Efficient Two-Photon Photorelease of Glycine by π-Extended Dipolar Coumarins. Chem. Sci. 2019, 10 (15), 4209–4219. 10.1039/C9SC00148D. 31057749 PMC6481246

[ref14] Shin J.; Verwilst P.; Choi H.; Kang S.; Han J.; Kim N. H.; Choi J. G.; Oh M. S.; Hwang J. S.; Kim D.; et al. Harnessing Intramolecular Rotation To Enhance Two-photon Imaging of Aβ Plaques through Minimizing Background Fluorescence. Angew. Chem., Int. Ed. 2019, 58 (17), 5648–5652. 10.1002/anie.201900549. 30809896

[ref15] Krystkowiak E.; Maciejewski A. Changes in Energy of Three Types of Hydrogen Bonds upon Excitation of Aminocoumarins Determined from Absorption Solvatochromic Experiments. Phys. Chem. Chem. Phys. 2011, 13 (23), 11317–11324. 10.1039/c1cp20767a. 21566823

[ref16] Krystkowiak E.; Dobek K.; Burdziński G.; Maciejewski A. Radiationless Deactivation of 6-Aminocoumarin from the S1-ICT State in Nonspecifically Interacting Solvents. Photochem. Photobiol. Sci. 2012, 11 (8), 1322–1330. 10.1039/c2pp25065a. 22622372

[ref17] Kamada K.; Iwase Y.; Sakai K.; Kondo K.; Ohta K. Cationic Two-Photon Absorption Chromophores with Double- and Triple-Bond Cores in Symmetric/Asymmetric Arrangements. J. Phys. Chem. C 2009, 113 (27), 11469–11474. 10.1021/jp901500v.

[ref18] Krzeszewski M.; Thorsted B.; Brewer J.; Gryko D. T. Tetraaryl-, Pentaaryl-, and Hexaaryl-1,4-Dihydropyrrolo[3,2- *b*]Pyrroles: Synthesis and Optical Properties. J. Org. Chem. 2014, 79 (7), 3119–3128. 10.1021/jo5002643. 24655027

[ref19] Tasior M.; Vakuliuk O.; Koga D.; Koszarna B.; Górski K.; Grzybowski M.; Kielesiński Ł.; Krzeszewski M.; Gryko D. T. Method for the Large-Scale Synthesis of Multifunctional 1,4-Dihydro-Pyrrolo[3,2-b]Pyrroles. J. Org. Chem. 2020, 85 (21), 13529–13543. 10.1021/acs.joc.0c01665. 32907329 PMC7656515

[ref20] Poronik Y. M.; Baryshnikov G. V.; Deperasińska I.; Espinoza E. M.; Clark J. A.; Ågren H.; Gryko D. T.; Vullev V. I. Deciphering the Unusual Fluorescence in Weakly Coupled Bis-Nitro-Pyrrolo[3,2-b]Pyrroles. Commun. Chem. 2020, 3 (1), 190 10.1038/s42004-020-00434-6. PMC9814504 36703353

[ref21] Kamada K.; Matsunaga K.; Yoshino A.; Ohta K. Two-Photon-Absorption-Induced Accumulated Thermal Effect on Femtosecond Z-Scan Experiments Studied with Time-Resolved Thermal-Lens Spectrometry and Its Simulation. J. Opt. Soc. Am. B 2003, 20 (3), 529–537. 10.1364/JOSAB.20.000529.

[ref22] Sheik-Bahae M.; Said A. A.; Wei T.-H.; Hagan D. J.; van Stryland E. W. Sensitive Measurement of Optical Nonlinearities Using a Single Beam. IEEE J. Quantum Electron. 1990, 26 (4), 760–769. 10.1109/3.53394.

[ref23] Kamada K.; Ohta K.; Iwase Y.; Kondo K. Two-Photon Absorption Properties of Symmetric Substituted Diacetylene: Drastic Enhancement of the Cross Section near the One-Photon Absorption Peak. Chem. Phys. Lett. 2003, 372 (3–4), 386–393. 10.1016/S0009-2614(03)00413-5.

[ref24] Nagakura S. Excited States. In Excited States; Lim E. C., Ed.; Academic: New York, 1975; pp 324–340.

[ref25] Liu X.; Cole J. M.; Xu Z. Substantial Intramolecular Charge Transfer Induces Long Emission Wavelengths and Mega Stokes Shifts in 6-Aminocoumarins. J. Phys. Chem. C 2017, 121 (24), 13274–13279. 10.1021/acs.jpcc.7b04176.

[ref26] Grabowski Z. R.; Rotkiewicz K.; Rettig W. Structural Changes Accompanying Intramolecular Electron Transfer: Focus on Twisted Intramolecular Charge-Transfer States and Structures. Chem. Rev. 2003, 103 (10), 3899–4032. 10.1021/cr940745l. 14531716

[ref27] Krauter C. M.; Möhring J.; Buckup T.; Pernpointner M.; Motzkus M. Ultrafast Branching in the Excited State of Coumarin and Umbelliferone. Phys. Chem. Chem. Phys. 2013, 15 (41), 17846 10.1039/c3cp52719k. 24045307

[ref28] Morawski O. W.; Kielesiński Ł.; Gryko D. T.; Sobolewski A. L. Highly Polarized Coumarin Derivatives Revisited: Solvent-Controlled Competition Between Proton-Coupled Electron Transfer and Twisted Intramolecular Charge Transfer. Chem. - Eur. J. 2020, 26 (32), 7281–7291. 10.1002/chem.202001079. 32212353

