# Supplementary material for: Quadrupolar Dyes Based on Highly Polarized Coumarins
Source: Org Lett. 2021 Aug 16;23(17):6770–4. doi: 10.1021/acs.orglett.1c02349 (PMC8419859; doi:10.1021/acs.orglett.1c02349)
Supplement: Supplementary file 1 — ol1c02349_si_001.pdf [file ol1c02349_si_001.pdf]

SUPORTING INFORMATION

Quadrupolar Dyes Based on Highly Polarized Coumarins

Krzysztof Górski,[a] Irena Deperasińska,[b] Glib V. Baryshnikov,[c] Suhei Ozaki,[d,e] Kenji Kamada,*[d,e] Hans Ågren*[f] and Daniel T. Gryko *[a]

[a] *Institute of Organic Chemistry, Polish Academy of Sciences, Kasprzaka 44/52, 01-224 Warsaw, Poland.*

[b] *Institute of Physics, Polish Academy of Sciences, Al. Lotników 32/46, 02-668 Warsaw, Poland.*

[c] *Department of Physics and Astronomy, Uppsala University, Box 516, SE-751 20 Uppsala, Sweden*

[d] *NMRI, National Institute of Advanced Industrial Science and Technology (AIST), Ikeda, Osaka 563-8577, Japan*

[e] *Department of Chemistry, Graduate School of Science and Technology, Kwansei Gakuin University, Sanda 669-1337, Japan*

[f] *Laboratory of Organic Electronics, Department of Science and Technology, Linköping University, SE-60174 Norrköping, Sweden*

Table of Contents

1. Quantum chemical calculations results: S2

2. Experimental: S12

3. General procedure for the synthesis of formylcoumarins 2: S12

4. General procedure for the synthesis of Coum6 and Coum7: S12

5. 500 MHz NMR spectra of 2a in $CDCl_3$ S13

6. 500 MHz NMR spectra of 2b in $CDCl_3$ S15

7. 500 MHz NMR spectra of Coum6 in $CDCl_3$ S17

8. 500 MHz NMR spectra of Coum7 in $CDCl_3$ S19

9. Two-photon absorption measurements of Coum6 and Coum7: S21

1. **Quantum chemical calculations results:**

a. **Computational details**

Quantum chemical calculations were performed using the Gaussian 16 package.[S1] The structures of isolated molecules in their electronic ground (S_0) and excited singlet states (S_1) were optimized with the aid of the DFT, TD DFT B3LYP/6-31G(d,p) and CAM-B3LYP/6-31G(d,p) methods and were confirmed by positive values of all calculated vibrations frequencies. The optimisation of S_0 structures and calculations of $S_0 \rightarrow S_i$ transition energies were performed also by B3LYP/cc-pVTZ method. Solvent effect was introduced within the PCM procedure.

Simulation of two-photon absorption (TPA) was performed in Osaka by using the Gaussian 09 package.[S2] The paramters needed fo the calculations (i.e., transition energies, permanent dipole moments in excited states, and transition dopole moments between the excited states) were calculated by using the Tamm–Dancoff approximation (TDA)[S3] was used for the same structure as used in the calculations above. The solvent effect was also introduced (PCM, for toluene). The TPA spectrum was simulated based on the reported method[S4] with the empirical relaxation constant (0.1 eV).

[S1] Gaussian 16, Revision A.03, M. J. Frisch, G. W. Trucks, H. B. Schlegel, G. E. Scuseria, M. A. Robb, J. R. Cheeseman, G. Scalmani, V. Barone, G. A. Petersson, H. Nakatsuji, X. Li, M. Caricato, A. V. Marenich, J. Bloino, B. G. Janesko, R. Gomperts, B. Mennucci, H. P. Hratchian, J. V. Ortiz, A. F. Izmaylov, J. L. Sonnenberg, D. Williams-Young, F. Ding, F. Lipparini, F. Egidi, J. Goings, B. Peng, A. Petrone, T. Henderson, D. Ranasinghe, V. G. Zakrzewski, J. Gao, N. Rega, G. Zheng, W. Liang, M. Hada, M. Ehara, K. Toyota, R. Fukuda, J. Hasegawa, M. Ishida, T. Nakajima, Y. Honda, O. Kitao, H. Nakai, T. Vreven, K. Throssell, J. A. Montgomery, Jr., J. E. Peralta, F. Ogliaro, M. J. Bearpark, J. J. Heyd, E. N. Brothers, K. N. Kudin, V. N. Staroverov, T. A. Keith, R. Kobayashi, J. Normand, K. Raghavachari, A. P. Rendell, J. C. Burant, S. S. Iyengar, J. Tomasi, M. Cossi, J. M. Millam, M. Klene, C. Adamo, R. Cammi, J. W. Ochterski, R. L. Martin, K. Morokuma, O. Farkas, J. B. Foresman, and D. J. Fox, Gaussian, Inc., Wallingford CT, 2016.

[S2] Gaussian 09, Revision D.01, M. J. Frisch, G. W. Trucks, H. B. Schlegel, G. E. Scuseria, M. A. Robb, J. R. Cheeseman, G. Scalmani, V. Barone, B. Mennucci, G. A. Petersson, H. Nakatsuji, M. Caricato, X. Li, H. P. Hratchian, A. F. Izmaylov, J. Bloino, G. Zheng, J. L. Sonnenberg, M. Hada, M. Ehara, K. Toyota, R. Fukuda, J. Hasegawa, M. Ishida, T. Nakajima, Y. Honda, O. Kitao, H. Nakai, T. Vreven, J. A. Montgomery, Jr., J. E. Peralta, F. Ogliaro, M. Bearpark, J. J. Heyd, E. Brothers, K. N. Kudin, V. N. Staroverov, T. Keith, R. Kobayashi, J. Normand, K. Raghavachari, A. Rendell, J. C. Burant, S. S. Iyengar, J. Tomasi, M. Cossi, N. Rega, J. M. Millam, M. Klene, J. E. Knox, J. B. Cross, V. Bakken, C. Adamo, J. Jaramillo, R. Gomperts, R. E. Stratmann, O. Yazyev, A. J. Austin, R. Cammi, C. Pomelli, J. W. Ochterski, R. L. Martin, K. Morokuma, V. G. Zakrzewski, G. A. Voth, P. Salvador, J. J. Dannenberg, S. Dapprich, A. D. Daniels, O. Farkas, J. B. Foresman, J. V. Ortiz, J. Cioslowski, and D. J. Fox, Gaussian, Inc., Wallingford CT, 2013.

[S3] S. Hirata, M. Head-Gordon, *Chem. Phys. Lett.*, **1999**, *314*, 291–299.

[S4] K. Ohta, S. Yamada, K. Kamada, A. D. Slepkov, F. A, Hegmann, R. R. Tykwinski, L. D. Shirtcliff, M. M. Haley, P. Sałek, F. Gel'mukhanov, H. Ågren, *J. Phys. Chem. A* **2011**, *115*, 105–117.

b. **Electronic spectra**

Simulations of the **Coum6** and **Coum7** electronic absorption spectra are shown in Fig. S1. These simulations are based on the results of the TD DFT B3LYP/6-31G(d,p) calculations of the electronic transitions energies and oscillator strengths for both molecules (detailed in Table S1). The spectra shown in Fig. S1 correspond to the molecules optimized in the C_i symmetry but can be considered as sufficiently representative due to the similarity of the results obtained in C_i and C_2 symmetries. This is shown in Table S2, where the results of the calculations of the transition energies and the oscillator strengths for the absorption and emission of **Coum6** and **Coum7** in both symmetries, isolated and in various solvents are collected.

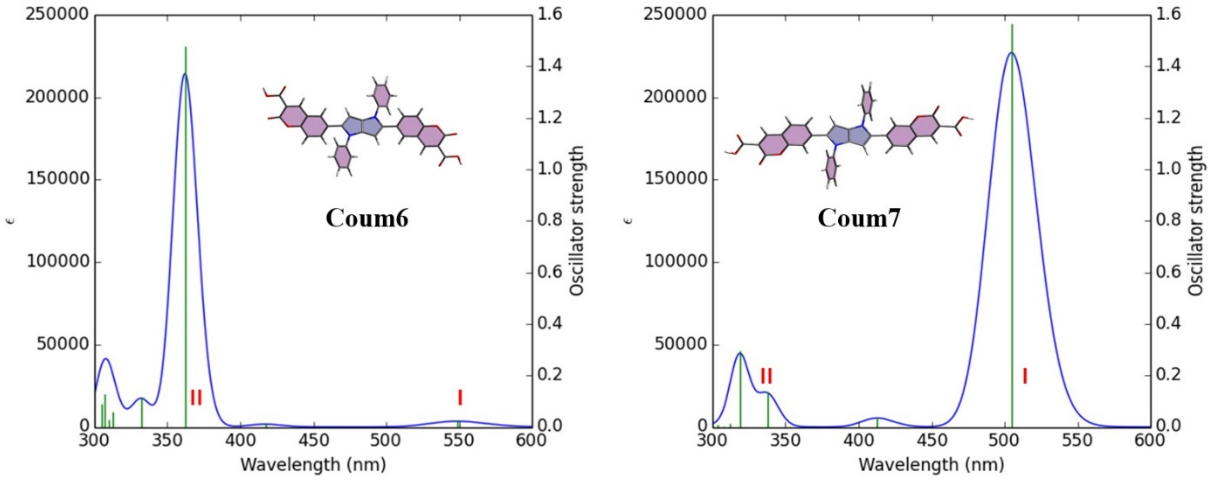

Fig. S1 Simulation of the electronic absorption spectra of isolated **Coum6** and **Coum7** molecules. This simulation is based on the results of TD DFT B3LYP/6-31G(d,p) calculations shown in detail in Table S1.

The obtained computational results for such similar molecules as **Coum6** and **Coum7** indicate surprisingly large differences in the energy (E) and intensity (f) of the $S_0 \rightarrow S_1$ transition (marked as **I** in the spectra shown in Fig. S1.) This observation can be described by the relations:

E($S_0 \rightarrow S_1$) for **Coum7** > E($S_0 \rightarrow S_1$) for **Coum6** (1)

f($S_0 \rightarrow S_1$) for **Coum7** >> f($S_0 \rightarrow S_1$) for **Coum6** (2)

These relations are preserved when the functional basis in the calculations is changed (Table S2) or the polarity of the medium is changed (Table S3).

Due to the complexity of the **Coum6** and **Coum7** molecules, we performed additional calculations (with the structures optimization) to determine if such relations would be maintained for simpler systems. Part B of Table S4 presents the results for the **Coum6** and **Coum7** systems devoid of aromatic substituents. Obtained results suggests that the aromatic substituents practically do not affect the optical properties of the **Coum6** and **Coum7**.

Table S1 The calculated wavelengths [in nm] and oscillator strengths of the $S_0 \rightarrow S_i$ absorption of isolated **Coum6** and **Coum7** molecules (TD DFT B3LYP/6-31G(d,p)).

$S_0 \rightarrow S_i$	Coum6			Coum7			$S_0 \rightarrow S_i$	Coum6			Coum7		
no	λ [nm]	f	sym	λ [nm]	f	sym	no	λ [nm]	f	sym	λ [nm]	f	sym
**1**	548.77	0.0240	AU	504.84	1.5666	AU	**9**	312.41	0.0599	AU	312.28	0.0121	AU
**2**	548.72	0.0000	AG	442.47	0.0000	AG	**10**	310.69	0.0000	AG	310.58	0.0000	AG
**3**	417.34	0.0000	AG	412.96	0.0374	AU	**11**	310.15	0.0304	AU	308.97	0.0000	AG
**4**	417.16	0.0117	AU	374.70	0.0000	AG	**12**	308.03	0.0000	AG	308.15	0.0010	AU
**5**	362.12	1.4783	AU	344.94	0.0000	AG	**13**	307.41	0.1291	AU	307.76	0.0000	AG
**6**	332.64	0.0000	AG	337.75	0.1365	AU	**14**	305.25	0.0915	AU	303.27	0.0102	AU
**7**	332.05	0.1184	AU	321.33	0.0000	AG	**15**	302.92	0.0000	AG	300.32	0.0000	AG
**8**	323.25	0.0000	AG	318.91	0.2977	AU							

Table S2. The wavelengths [in nm] and oscillator strengths of the absorption and fluorescence of **Coum6** and **Coum7** molecules calculated in different basis sets.

			B3LYP/6-31G(d,p)		CAM-B3LYP/6-31G(d,p)		B3LYP/cc-pVTZ		B3LYP-37/cc-pVTZ		exp
			λ [nm]	f	λ [nm]	f	λ [nm]	f	λ [nm]	f	λ [nm]
**Coum6**	abs I	$S_0 \rightarrow S_1$	548.7	0.0240	360.9	0.1147	553.9	0.0180	448	0.0621	439-470
	abs II	$S_0 \rightarrow S_5$	362.1	1.4783	308.3	1.6501	367.7	1.3340	342	1.9141	370
	flu	$S_1 \rightarrow S_0$	678.9	0.0171	427.8	0.1108			598	0.0205	641
**Coum7**	abs	$S_0 \rightarrow S_1$	505.0	1.5487	392.5	1.882	509.8	1.5807	475	2.0885	486
	flu	$S_1 \rightarrow S_0$	548.7	1.3658	455.4	2.2179			535	2.4413	574

Table S3 The calculated wavelengths [in nm] and oscillator strengths of the absorption and fluorescence of **Coum6** and **Coum7** molecules, isolated and in solvents (TD DFT B3LYP/6-31G(d,p)).

		absorption				fluorescence		absorption				fluorescence	
		Band I		Band II				Band I		Band II			
solvent	symm	λ [nm]	f	λ [nm]	f	λ [nm]	f	λ [nm]	f	λ [nm]	f	λ [nm]	f
		**Coum6**						**Coum7**					
isol	C_i	548.8	0.024	362.1	1.478	679.5	0.000	504.9	1.567			542.2	1.480
	C_2	546.7	0.020	364.5	1.437	678.9	0.017	505.0	1.549			548.7	1.366
CHX	C_i	565.0	0.020	371.5	1.716	707.4	0.023	537.0	1.787			582.1	1.892
	C_2	563.1	0.024	374.6	1.690	707.2	0.020	540.5	1.807			585.7	1.814
ACN	C_i	575.7	0.041	382.2	1.920	743.8	0.030	576.2	1.994			658.8	2.336
	C_2	573.8	0.032	384.6	1.887	743.2	0.023	576.3	1.973			660.9	2.289

Table S4 Energies ([nm], [eV]) and oscillator strengths of the $S_0 \rightarrow S_1$ transition for molecules similar to **Coum6** and **Coum7**

	**Coum6**	**Coum7**
**A**	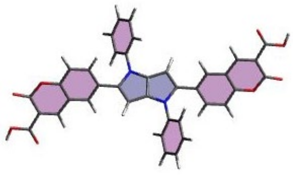	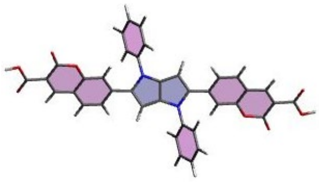
$E(S_0 \rightarrow S_1)$ [nm] f	548.8 / 0.024	504.9 / 1.567
**B**	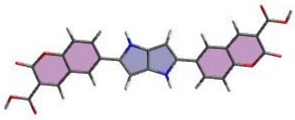	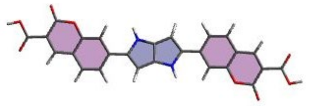
$E(S_0 \rightarrow S_1)$ [nm] f	578.0 / 0.020	510.5 / 1.882
**C**	**a-Coum6** 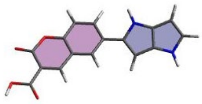	**a-Coum7** 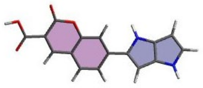
$E(S_0 \rightarrow S_1)$ [nm]	585.7 / 0.010	450.4 / 0.804
$\mu(S_0)$	6.1 D	7.7 D
$\mu(S_1)$	23.9 D	16.4 D

Part C of Table S4 shows the calculation results for the asymmetric systems **a-Coum6** and **a-Coum7** where **Coum6** and **Coum7** have been stripped of one coumarin unit. In this manner, we check whether the results obtained for starting molecules do not hide unexpected effects. As can be seen from Table S4C, also in the case of asymmetric molecules, the $S_0 \rightarrow S_1$ transition energy and its oscillator strength corresponding to **a-Coum6** are smaller compared to **a-Coum7**, i.e. the relations established for the parent D-A systems are recreated. The dipole moments of these nonsymmetric molecules are non-zero. It follows from Table S4C that the dipole moments of both molecules in the ground state are comparable, while in the S_1 excited state the **a-Coum6** dipole moment is much greater than in the case **a-Coum7**.

c. Energy diagram of the Coum6 and Coum7

Fig. S2 shows the energy of **Coum6** and **Coum7** molecular orbitals as well as electron density distribution of orbitals, participating in the lowest energy electronic transitions. In the center of the Fig. S2 pyrrolo[3,2-*b*]pyrrole and coumarin orbitals are located. Blue arrows show the intense electron transitions: $S_0 \rightarrow S_1$ in **Coum7** (Band I in Table S1-S4) and $S_0 \rightarrow S_5$ in **Coum6** (Band II in Table S1-S4). In both cases discussed transitions possess CT nature and the electron density of the final-state is delocalized over the entire molecule.

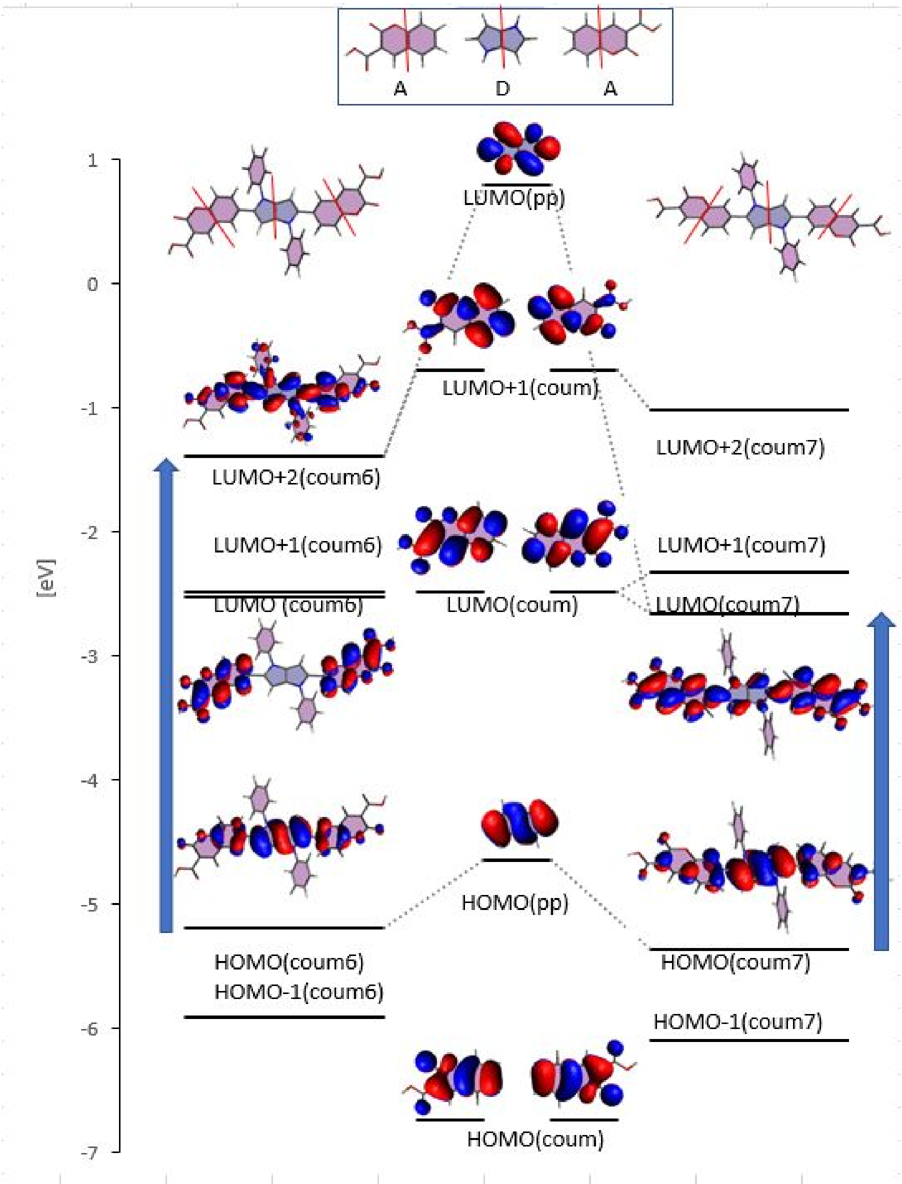

Fig. S2 **Coum6** and **Coum7** energy levels diagram.

d. Charge-transfer character of S_1 state of Coum6 and Coum7

The CT nature of the $S_0 \rightarrow S_1$ transition is apparent for the symmetrical, dipoleless **Coum6** and **Coum7** molecules, as illustrated in Fig. S2. Charge transfer from pyrrolo[3,2-*b*]pyrrole core to coumarin units takes place in both molecules but is greater in the case of **Coum6**. This means stronger Coulomb interactions in the excited state of **Coum6**, which is consistent and explains the first of the discussed inequalities (1). The well-known expression describes the CT transition energy as:

$$E_{CT} = I_D - E_A - C \qquad (3)$$

where E_{CT} is the CT transition energy, I_D - donor ionization potential, E_A - acceptor electron affinity, and C - Coulomb interactions between donor and acceptor in CT state. With the same I_D and E_A values for both molecules, the higher C value for **Coum6** leads to a lower E_{CT} value for **Coum6** compared to the **Coum7**. Trying to explain the difference in oscillator strengths for $S_0 \rightarrow S_1$ transitions in **Coum6** and **Coum7** you can recall the simplified expression for the transition moment $M_{HOMO \rightarrow LUMO}$:

$$M_{HOMO \rightarrow LUMO} = < \Psi_{HOMO} | R | \Psi_{LUMO} > \; \Rightarrow \sum_k c_k(HOMO)\, c_k(LUMO) R_k \qquad (4)$$

where $c_k(HOMO)$ and $c_k(LUMO)$ are LCAO coefficients for HOMO and LUMO orbitals and R_k is the position vector of atom *k*. Putting this expression together with the shapes of HOMO and LUMO the orbitals becomes clear that there is no contribution to the transition moment from the atoms of the central pyrrolo[3,2-*b*]pyrrole unit of the molecule in the case of **Coum6**, because and $c_k(LUMO)$ for these atoms are zero. On the other hand, there is such contribution in the case of **Coum7**, and thus creates the second inequality we have discussed, f($S_0 \rightarrow S_1$) for **Coum7** >> f($S_0 \rightarrow S_1$) for **Coum6**. The results presented so far indicate that the source of the described differentiation properties of both molecules should be sought in the differences between the connections of pyrrolo[3,2-*b*]pyrrole units with coumarin in positions 6 and 7.

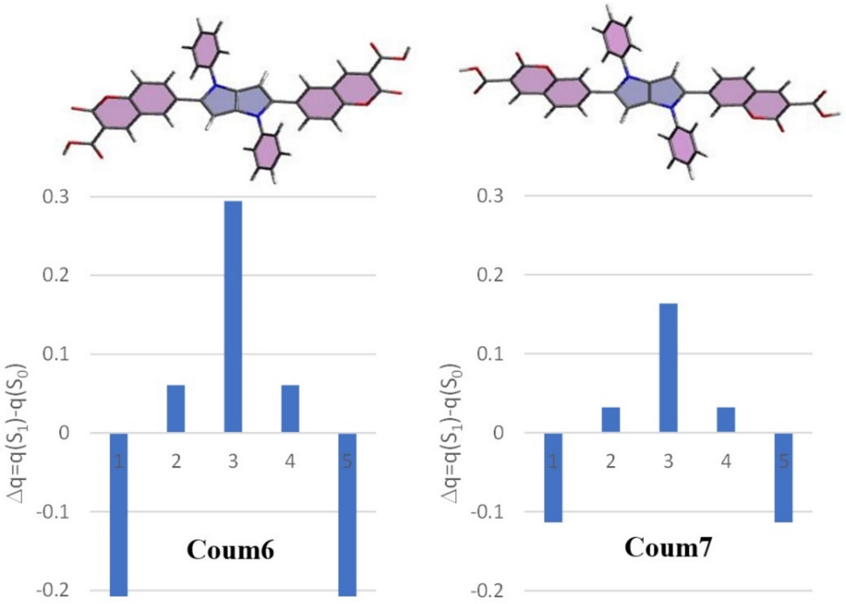

Fig. S3 Changes in the electron charge distribution due to electronic excitation $S_0 \rightarrow S_1$. $\Delta q = q(S_1) - q(S_0)$ where $q(S_x)$ is net electronic charge in S_x state, marking: 1 and 5 for coumarin, 2 and 4 for phenyl, 3 for pyrrolo[3,2-b]pyrrole core.

e. Structural differences between Coum6 and Coum7

In Table S5 the presented structural differences between **Coum6** and **Coum7** (using the example of their asymmetric representatives) results from the rotation of the coumarin subunit around an axis perpendicular to its plane by 60^0. Maintaining the geometrical order allows for interpretation of the HOMO and LUMO shapes of **a-Coum6** and **a-Coum7** in terms of the component orbitals combination (Tables S6 and S7).

Table S5 Structural differences between D-A systems, **a-Coum6** and a-**Coum7**, as the result of coumarin subunit rotation around an axis perpendicular to its plane by 60^0.

	pyrrolo[3,2-b]pyrrole core		coumarin		D-A system
**a-Coum6**	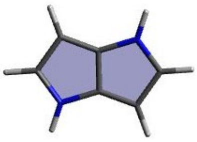	+	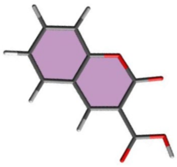	=>	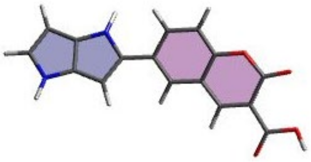
**a-Coum7**	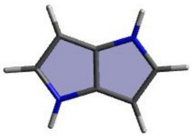	+	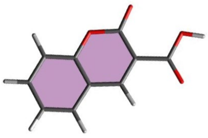	=>	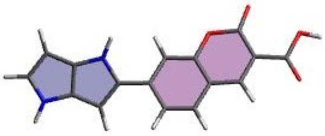

Table S6 HOMO of D-A systems, **a-Coum6** and **a-Coum7**, as a composite of the HOMO orbitals of their components (the coumarin HOMO orbital in both cases is the same orbital but rotated by 60^0 – compare Table S5)

	pyrrolo[3,2-b]pyrrole core		coumarin		D-A system
**a-Coum6**	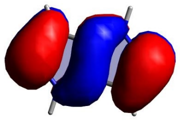	+	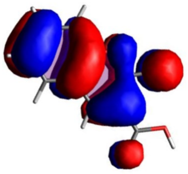	=>	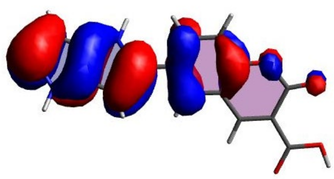
**a-Coum7**	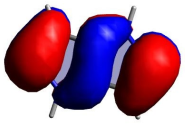	+	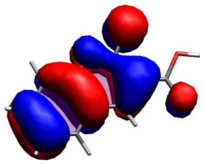	=>	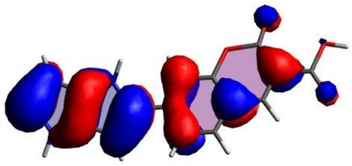

Conclusion: in both cases, $\Psi(S_0)\approx a\psi^{pp}_{HOMO}+b\psi^{cum}_{HOMO}$ (a > b)

Table S7 LUMO of D-A systems, **a-Coum6** and **a-Coum7**, as a composite of the LUMO orbitals of their components (the coumarin LUMO orbital in both cases are the same orbital but rotated by 60^0 – compare Table S5).

	pyrrolo[3,2-b]pyrrole core		coumarin		D-A system
**a-Coum6**		+	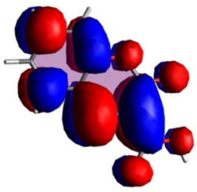	=>	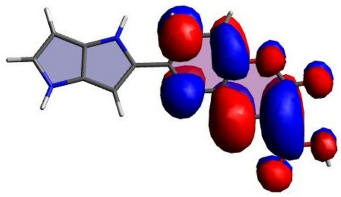
**a-Coum7**	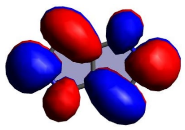	+	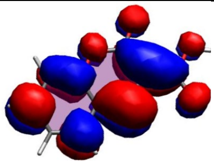	=>	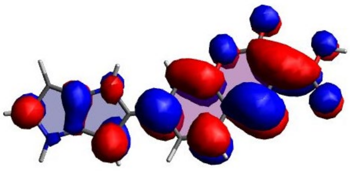

Conclusion: for **a-Coum6** $\Psi(S_1)\approx\psi^{cum}_{LUMO}$, while for **a-Coum7**, $\Psi(S_1)\approx a^*\psi^{pp}_{LUMO}+b^*\psi^{cum}_{LUMO}$ ($a^* < b^*$)

The presented pictorial story (using the example of **a-Coum6** and **a-Coum7**) recreates the properties of the HOMO and LUMO orbitals of **Coum6** and **Coum7** molecules. In particular, you can see that LUMO of **a-Coum6** (as opposed to **a-Coum7**) is practically the same as LUMO of coumarin. This difference can be explained in terms of local symmetry of orbital with respect to the axis with bond connecting pyrrolo[3,2-b]pyrrole and coumarin units

(Fig. S4). In the case of the connection via 7 position of coumarin, the orbitals on both components have the same symmetry which means overlap between both orbitals. On the other hand, the opposite effect is observed in the case of the connection via 6 position.

$$S(LUMO_{pp}, LUMO_{coum}) > 0 \text{ for } \mathbf{Coum7} \quad (5)$$

$$S(LUMO_{pp}, LUMO_{coum}) \approx 0 \text{ for } \mathbf{Coum6} \quad (6)$$

Referring to the theory of EDA systems, there is no short-range coupling between the charge distributions on donor and acceptor for **Coum6,** i.e. excitation leads to the separation of charges, determined by the potential difference between donor and acceptor and interacting by Coulomb forces. Contrary, at the connection by position 7 non-zero coupling extends charge distribution over the whole molecule.

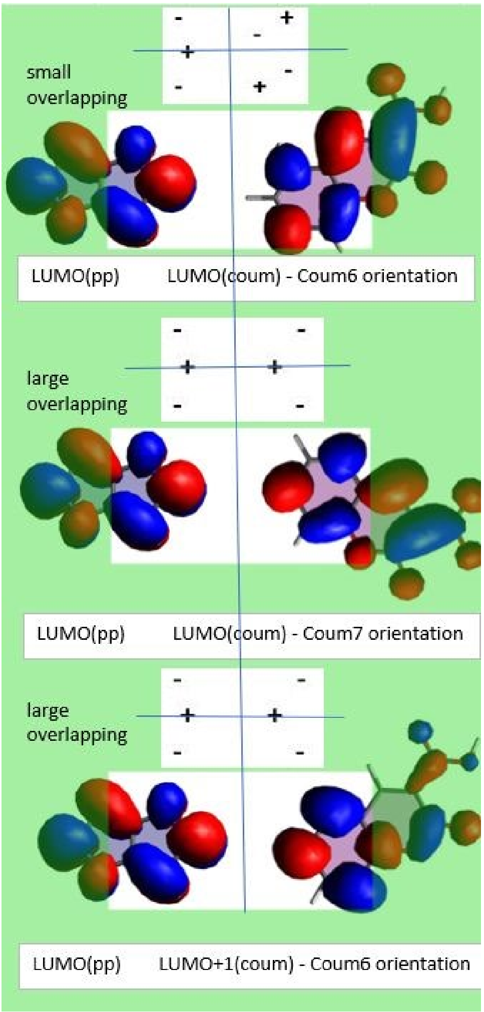

Fig. S4 *(On top and in the middle)* Mutual orientation of the LUMO orbitals of the pyrrolo[3,2-b]pyrrole unit and coumarin at connections through the 6 and 7 of the coumarin atoms. The local symmetry element is the axis containing the bond between the two units. In this local symmetry, the LUMO orbitals of both units have the same symmetry when linked through the 7-coumarin atom. This creates the conditions for interaction between the two charge distributions and its extension as shown in Table S7. The opposite effect is observed in the case of a connection through atom 6. (*At the bottom*) Overlapping of pyrrolo[3,2-b]pyrrole LUMO with LUMO+1 of coumarin in **Coum6**.

The **Coum6** strong absorption band shown in Fig. S1 (marked **II**) corresponds to the CT excitation in which the (LUMO+1) of coumarin is the acceptor orbital. In junction 6, unlike the LUMO, its symmetry is in line with the symmetry of the donor LUMO – see Fig. S4.

f. Two-photon absorption spectrum

Simulations of TPA spectra of **Coum6** and **Coum7** are shown in Fig. S5a-b. These simulations are based on the results of the TDA[S3] B3LYP/6-31G(d,p) calculations (PCM=toluene). Relaxation constant was chosen to be 0.1 eV for both one- and two-photon resonances. For **Coum6**, please note that the order of S_3 and degenerated pair of S_4 and S_5 in TD-DFT calculation are interchanged in TDA calculation by lowering the energy level of S_3 (342 nm → 332 nm). Thus, S_3, S_4, and S_5 in TDA correspond to S_4, S_5, and S_3 in TD-DFT respectively. Other states are in the same order for both calculations.

The intense TPA transition of **Coum6** was $S_0 \rightarrow S_8$ (Fig. S5a) mostly via S_5 (i.e., S_3 in TD-DFT) as the major intermediate state while that of **Coum7** was $S_0 \rightarrow S_2$ (Fig. S5b) mostly via S_1 as the major intermediate state. The corresponding orbital transitions are HOMO → LUMO+2 → LUMO+3 (**Coum6**) and HOMO → LUMO → LUMO+1 (**Coum7**). HOMOs (**Coum6, Coum7**), LUMO+2 (**Coum6**), and LUMO (**Coum7**) were shown in Fig. S2 above. The orbital pattern of LUMO+3 (**Coum6**) and LUMO+1(**Coum7**) (Fig. S5c-d) showed large overlap with LUMO+2 (**Coum6**) and LUMO (**Coum7**), respectively, for wide area, resulting in large transition dipole moments between the excited states (13.6 D for $S_5 \rightarrow S_8$ (**Coum6**) and 25.5 D for $S_1 \rightarrow S_2$ (**Coum7**)) and strong TPA transitions.

(a)

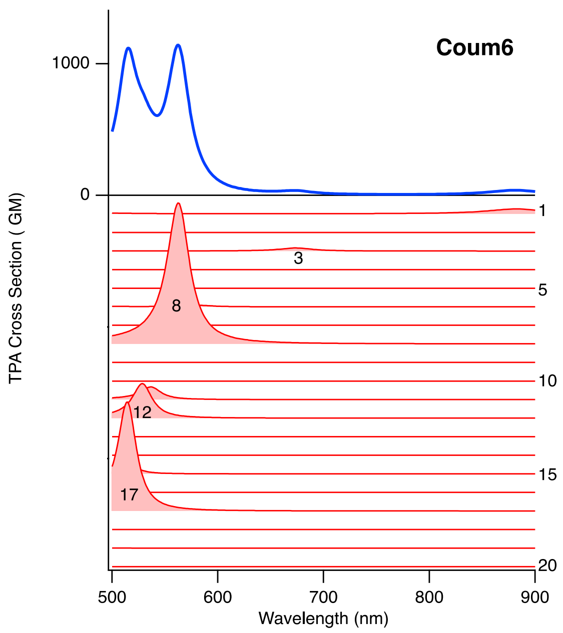

(b)

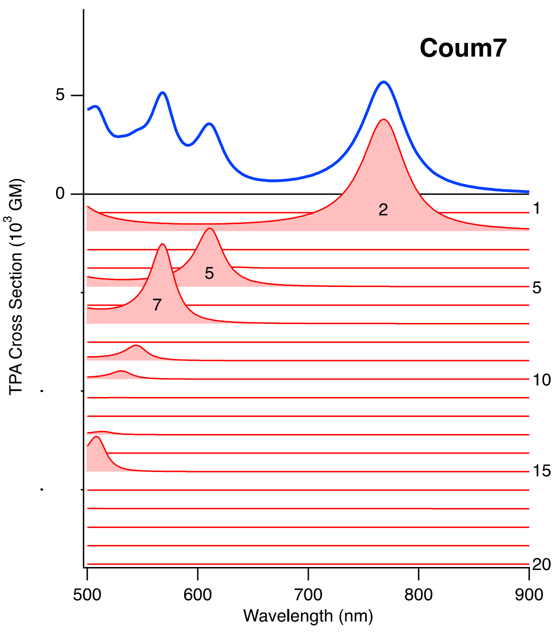

(c)

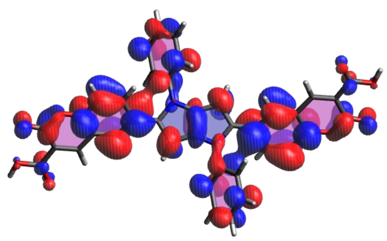

(d)

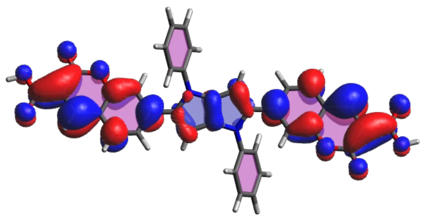

Fig. S5 Simulation of two-photon absorption spectra (blue curve) of (a) **Coum6** and (b) **Coum7** in toluene and their decomposed spectral components by the destination excites states (red curves with fill). The top is $S_0 \rightarrow S_1$ and the bottom is $S_0 \rightarrow S_{20}$ TPA transition. The number (n) means the destination excited state is S_n. Orbital pattern of (c) LUMO+3 of **Coum6** and (d) LUMO +1 of **Coum7**.

g. XYZ coordinates

Coum6				Coum7			
E(RB3LYP) =	-2172.65918732	A.U.		E(RB3LYP) =	-2172.66376239	A.U.	
H	1.8622	10.7772	1.9200	H	0.4437	11.5624	-1.4726
O	-0.7888	9.7625	-0.3997	O	0.0915	10.6602	-1.4987
O	1.2214	10.3216	1.3537	O	1.9480	10.3702	-0.2837
O	2.3423	8.5874	2.2159	C	0.9561	9.9066	-0.8015
C	1.4671	9.0109	1.4951	C	0.6000	8.4637	-0.7159
C	-0.4822	8.6278	-0.1846	O	-1.4393	8.4547	-1.9847
C	0.5773	8.1186	0.6966	H	2.3264	8.1233	0.4259
O	-1.2227	7.6401	-0.8537	C	-0.6000	7.8981	-1.3412
C	0.7888	6.7835	0.8173	C	1.4518	7.6647	-0.0179
C	-1.0022	6.3107	-0.7254	O	-0.7760	6.5154	-1.1497
H	-2.5774	5.8330	-2.0910	C	1.2296	6.2727	0.1386
H	1.5806	6.4531	1.4775	H	2.9732	5.8078	1.3076
C	0.0108	5.8221	0.1098	C	0.0779	5.7298	-0.4512
C	-1.8082	5.4348	-1.4452	C	2.0845	5.4039	0.8406
C	0.1967	4.4364	0.2189	H	-3.7631	4.1114	2.8210
C	-1.5976	4.0745	-1.3312	C	-0.2223	4.3798	-0.3544
H	-4.9577	2.7852	1.7842	C	1.7934	4.0661	0.9468
H	0.9643	4.0650	0.8846	H	-1.1077	4.0156	-0.8509
H	-2.2123	3.4031	-1.9129	C	-3.6079	3.4717	1.9629
C	-0.5918	3.5421	-0.4959	H	-5.6326	3.6418	1.2675
H	-2.5232	2.5520	1.4539	C	0.6305	3.5239	0.3490
C	-4.5830	2.1760	0.9728	H	-1.5419	3.1029	2.4288
C	-3.2122	2.0531	0.7881	H	2.4456	3.4175	1.5130
H	-6.5360	1.6028	0.2865	C	-4.6591	3.2049	1.0928
C	-5.4695	1.5095	0.1346	C	-2.3563	2.9113	1.7450
C	-0.3245	2.1048	-0.4181	C	0.3909	2.0916	0.4463
H	1.8613	2.0236	-0.4667	C	-4.4524	2.3755	-0.0031
C	-2.7199	1.2728	-0.2566	C	-2.1488	2.0928	0.6373
C	0.9235	1.4987	-0.4268	H	-5.2637	2.1668	-0.6871
N	-1.3216	1.1172	-0.4554	C	-3.1992	1.8235	-0.2369
C	-4.9765	0.7196	-0.8975	N	-0.8780	1.4913	0.4050
C	-3.6072	0.6048	-1.0997	H	2.4286	1.2776	0.4136
H	-5.6583	0.1980	-1.5556	C	1.3691	1.0990	0.4489
H	-3.2198	0.0079	-1.9136	H	-3.0263	1.1955	-1.1000
C	0.6846	0.1096	-0.4870	C	-0.6844	0.1256	0.3858
H	-1.8622	-10.7772	1.9200	H	-0.4437	-11.5624	-1.4726
O	0.7888	-9.7625	-0.3997	O	-0.0915	-10.6602	-1.4987
O	-1.2214	-10.3216	1.3537	O	-1.9480	-10.3702	-0.2837
O	-2.3423	-8.5874	2.2159	C	-0.9561	-9.9066	-0.8015
C	-1.4671	-9.0109	1.4951	C	-0.6000	-8.4637	-0.7159
C	0.4822	-8.6278	-0.1846	O	1.4393	-8.4547	-1.9847
C	-0.5773	-8.1186	0.6966	H	-2.3264	-8.1233	0.4259
O	1.2227	-7.6401	-0.8537	C	0.6000	-7.8981	-1.3412

C	-0.7888	-6.7835	0.8173	C	-1.4518	-7.6647	-0.0179
C	1.0022	-6.3107	-0.7254	O	0.7760	-6.5154	-1.1497
H	2.5774	-5.8330	-2.0910	C	-1.2296	-6.2727	0.1386
H	-1.5806	-6.4531	1.4775	H	-2.9732	-5.8078	1.3076
C	-0.0108	-5.8221	0.1098	C	-0.0779	-5.7298	-0.4512
C	1.8082	-5.4348	-1.4452	C	-2.0845	-5.4039	0.8406
C	-0.1967	-4.4364	0.2189	H	3.7631	-4.1114	2.8210
C	1.5976	-4.0745	-1.3312	C	0.2223	-4.3798	-0.3544
H	4.9577	-2.7852	1.7842	C	-1.7934	-4.0661	0.9468
H	-0.9643	-4.0650	0.8846	H	1.1077	-4.0156	-0.8509
H	2.2123	-3.4031	-1.9129	C	3.6079	-3.4717	1.9629
C	0.5918	-3.5421	-0.4959	H	5.6326	-3.6418	1.2675
H	2.5232	-2.5520	1.4539	C	-0.6305	-3.5239	0.3490
C	4.5830	-2.1760	0.9728	H	1.5419	-3.1029	2.4288
C	3.2122	-2.0531	0.7881	H	-2.4456	-3.4175	1.5130
H	6.5360	-1.6028	0.2865	C	4.6591	-3.2049	1.0928
C	5.4695	-1.5095	0.1346	C	2.3563	-2.9113	1.7450
C	0.3245	-2.1048	-0.4181	C	-0.3909	-2.0916	0.4463
H	-1.8613	-2.0236	-0.4667	C	4.4524	-2.3755	-0.0031
C	2.7199	-1.2728	-0.2566	C	2.1488	-2.0928	0.6373
C	-0.9235	-1.4987	-0.4268	H	5.2637	-2.1668	-0.6871
N	1.3216	-1.1172	-0.4554	C	3.1992	-1.8235	-0.2369
C	4.9765	-0.7196	-0.8975	N	0.8780	-1.4913	0.4050
C	3.6072	-0.6048	-1.0997	H	-2.4286	-1.2776	0.4136
H	5.6583	-0.1980	-1.5556	C	-1.3691	-1.0990	0.4489
H	3.2198	-0.0079	-1.9136	H	3.0263	-1.1955	-1.1000
C	-0.6846	-0.1096	-0.4870	C	0.6844	-0.1256	0.3858

h. Energetical characteristics of Coum6 and Coum7 computed by B3LYP/6-31g(d,p)

Table S8 Selected energetical characteristics of **Coum6** and **Coum7** ground state optimized structures computed by B3LYP/6-31g(d,p) method in gas phase, incl. total energy (Etot), zero-point energy correction (EZPE), relative energy (Erel), frequency of the first normal vibration (ω1) and the number of imaginary frequencies (Nimg) in vibrational spectrum.

Compound	E_{tot}, a.u.	E_{ZPE}, a.u.	$E_{tot} + E_{ZPE}$, a.u.	E_{rel}, kcal mole^{-1}	ω_1, cm^{-1} (N_{img})
**Coum6**	-2172.65878774	0.517584	-2172.141204	+2.9	10 (0)
**Coum7**	-2172.66333368	0.517587	-2172.145747	0	10 (0)

2. Experimental:

All chemicals were used as received unless otherwise noted. All used for reaction solvents were pure for analysis grade and were taken without further purification. **1a** was synthesized according to literature procedure[S5] while **1b** was bought from Molcore and 4-decylaniline from AlfaAesar. All reactions requiring heating were carried out using an oil bath. All reported NMR spectra were recorded on a 500 MHz spectrometer. Chemical shifts (δ ppm) for ^{1}H and ^{13}C NMR were determined with TMS as the internal reference; *J* values are given in Hz. UV-Vis absorption spectra were recorded in toluene, 2-methyltetrahydrofyrane and dimethylsulfoxide. In order to determine the fluorescence quantum yield 9,10-diphenylantracene (**for Coum6**) and coumarin 153 (**for Coum7**) were used as a quantum yield standard. Chromatography was performed on silica (Kieselgel 60, 200-400 mesh). Mass spectra were obtained with EI ion source and the EBE double-focusing geometry mass analyzer or spectrometer equipped with electrospray ion source with q-TOF type mass analyzer.

[S5] J. R. Stille, J. A. Ward, C. Leffelman and K. A. Sullivan, *Tetrahedron Lett.*, 1996, **37**, 9267–9270.

3. General procedure for the synthesis of formylcoumarins 2:

Dialdehyde **1** (901mg. 6mmol) was suspended in a mixture of 18ml of isopropanol and dimethyl malonate (0.72ml, d=1.15g/ml, 6mmol) and then heated up. The obtained clear solution was brought to room temperature followed by the addition of 0.075ml of pyrrolidine. As the reaction time progressed, a white precipitate is formed.

2a: After 24h white solid was filtrated and dried to yield 946mg (68%), mp 191°C. ^{1}H NMR (500 MHz, $CDCl_3$) δ: 10.07 (s, 1H), 8.64 (s, 1H), 8.19-8.16 (m, 2H), 7.52 (d, *J* = 8.4 Hz, 1H), 3.99 (s, 3H). ^{13}C {^{1}H} NMR (126 MHz, $CDCl_3$) δ: 189.5, 163.1, 158.6, 155.5, 148.2, 134.4, 133.1, 131.8, 119.3, 118.1, 117.9, 53.1. HRMS (EI): m/z calculated for $C_{12}H_8O_5$: 232.0366 [M^{+}]; found: 232.0367.

2b: After 4h white solid was filtrated and dried to yield 988mg (71%), mp 205 °C. ^{1}H NMR (500 MHz, $CDCl_3$) δ: 10.11 (s, 1H), 8.56 (s, 1H), 7.85 (d, *J* = 7.8 Hz, 1H), 7.82 (s, 1H), 7.79 (d, *J* = 7.8 Hz, 1H), 3.98 (s, 3H). ^{13}C {^{1}H} NMR (126 MHz, $CDCl_3$) δ: 190.2, 163.2, 156.1, 155.8, 147.5, 139.9, 138.7, 130.4, 124.5, 122.1, 118.0, 53.2. HRMS (EI): m/z calculated for $C_{12}H_8O_5$: 232.0366 [M^{+}]; found: 232.0369.

4. General procedure for the synthesis of Coum6 and Coum7:

In an open round-bottom flask, formylcoumarin **2** (1.39g, 6mmol) and 4-decylaniline (1.40g, 6mmol) were dissolved in a mixture of 4.5ml of toluene and 4.5ml of acetic acid. The obtained yellow solution was heated up to 50°C (precipitate may appear). After 1h in constant temperature butane-1,2-dione (0.26ml, d=0.9808g/ml, 3mmol) and catalytic amount of iron(III) chlorate(VII) monohydrate (63mg, 3%mol) were added. The mixture turns dark and after a while precipitate appears. After 24 hours of stirring at 50 °C under open air, the solution was cooled and the formed precipitate was filtered off. The crude product was purified via column chromatography using silica gel as a stationary phase and chloroform as eluent.

Coum6 was obtained as orange solid, yield 343mg (9%), mp 240 °C, Rf=0.25 ($CHCl_3/SiO_2$). ^{1}H NMR (500 MHz, $CDCl_3$) δ: 8.39 (s, 2H), 7.48 (dd, *J* = 8.7, 2.0 Hz, 2H), 7.41 (d, *J* = 1.9 Hz, 2H), 7.21 – 7.16 (m, 10H), 6.42 (s, 2H), 3.94 (s, 6H), 2.64 (t, *J* = 7.7, 4H), 1.67 – 1.61 (m, 4H), 1.37 – 1.24 (m, 28H), 0.88 (t, *J* = 6.9 Hz, 6H). ^{13}C {^{1}H} NMR (126 MHz, $CDCl_3$) δ: 163.8, 156.6, 153.6, 149.0, 141.4, 136.8, 134.2, 133.8, 132.1, 130.8, 129.4, 127.9, 125.1, 118.1, 117.8, 116.6, 95.0, 52.9, 35.5, 31.9, 31.3, 29.6, 29.6, 29.4, 29.3, 22.7, 14.1. HRMS (EI): m/z calculated for $C_{60}H_{66}N_2O_8$: 942.4814 [M^{+}]; found: 942.4818.

Coum7 was obtained as red solid, yield 648mg (17%), mp 246 °C, Rf=0.2 ($CHCl_3/SiO_2$). ^{1}H NMR (500 MHz, $CDCl_3$) δ: 8.48 (s, 2H), 7.41 (d, *J* = 8.2 Hz, 2H), 7.25 – 2.18 (m, 10H), 7.10 (s, 2H), 6.54 (s, 2H), 3.94 (s, 6H), 2.67 (t, *J* = 7.7 Hz 4H), 1.71 – 1.63 (m, 4H), 1.36 – 1.28 (m, 28H), 0.88 (t, *J* = 6.9 Hz, 6H). ^{13}C {^{1}H} NMR (126 MHz, $CDCl_3$) δ: 163.9, 157.0, 155.3, 148.8, 142.0, 139.6, 136.7, 135.6, 134.5, 129.6, 129.2, 125.2, 124.0, 116.0, 115.8, 114.4, 96.8, 52.8, 35.5, 31.9, 31.2, 29.6, 29.6, 29.5, 29.4, 29.3, 22.7, 14.1. HRMS (EI): m/z calculated for $C_{60}H_{66}N_2O_8$: 942.4814 [M^{+}]; found: 942.4833.

5. 500 MHz NMR spectra of 2a in CDCl₃

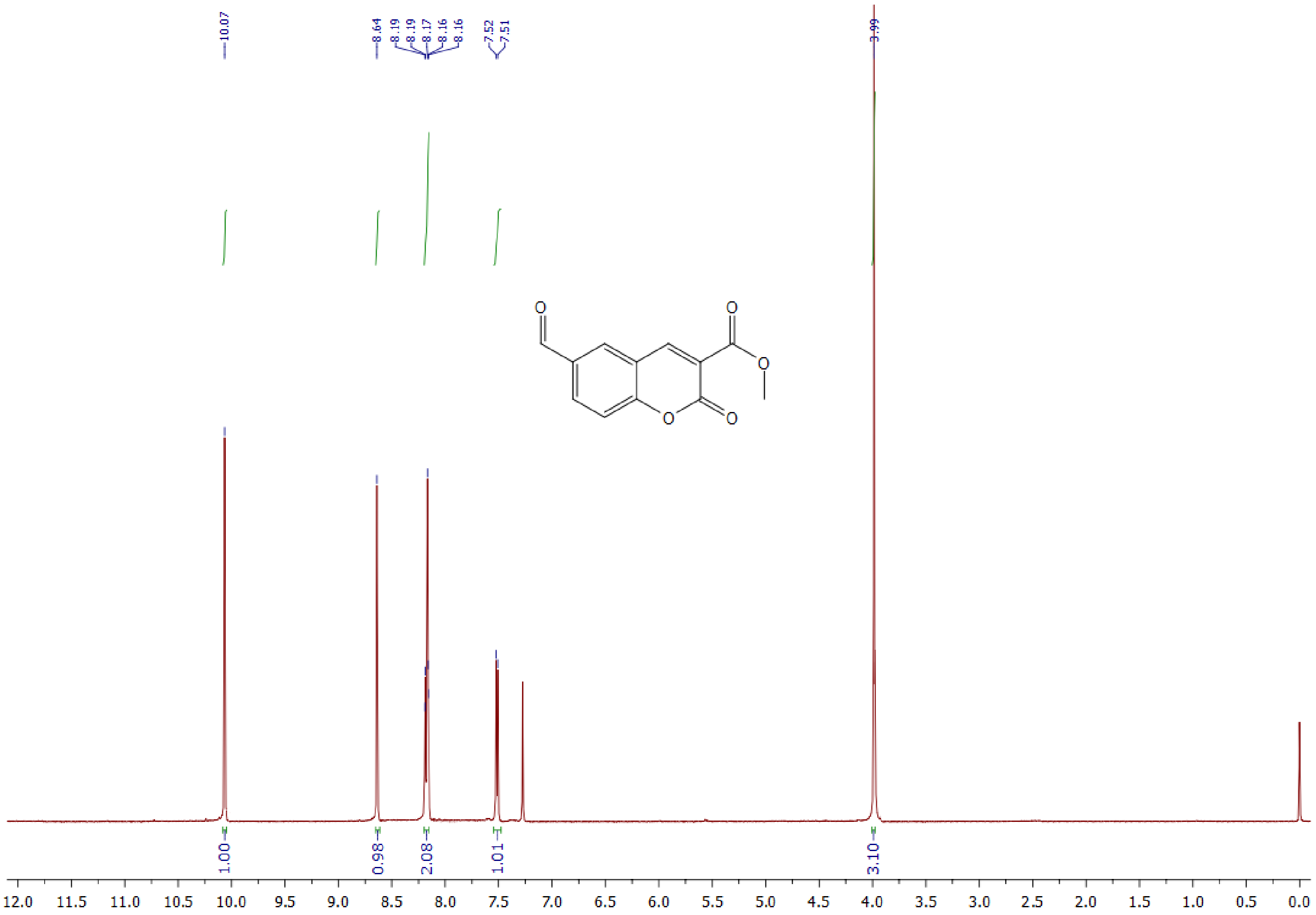

Fig. S6 500 MHz ^{1}H NMR spectrum of 2a in $CDCl_3$

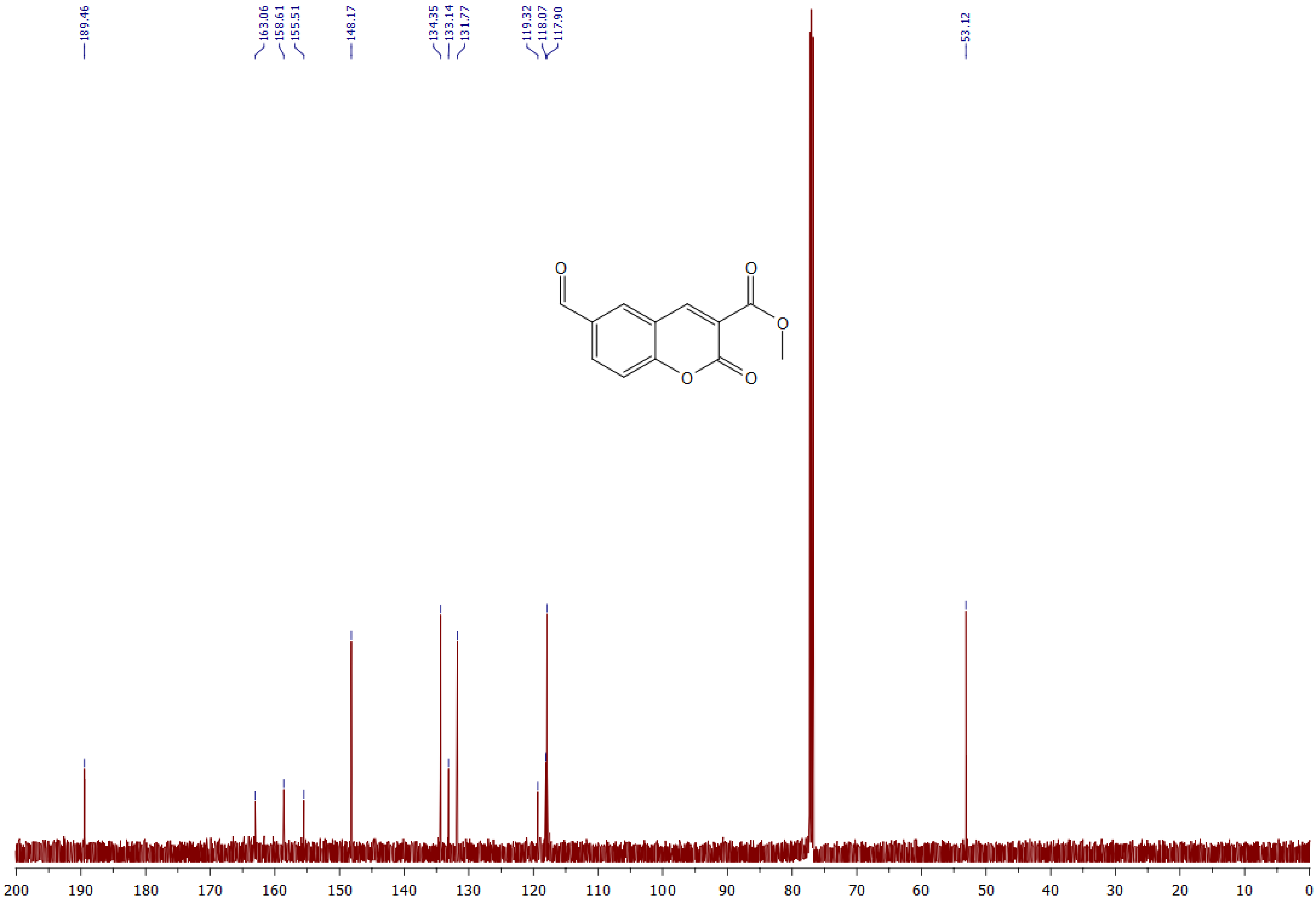

Fig. S7 500 MHz ^{13}C {^{1}H} NMR spectrum of 2a in $CDCl_3$

6. 500 MHz NMR spectra of 2b in CDCl₃

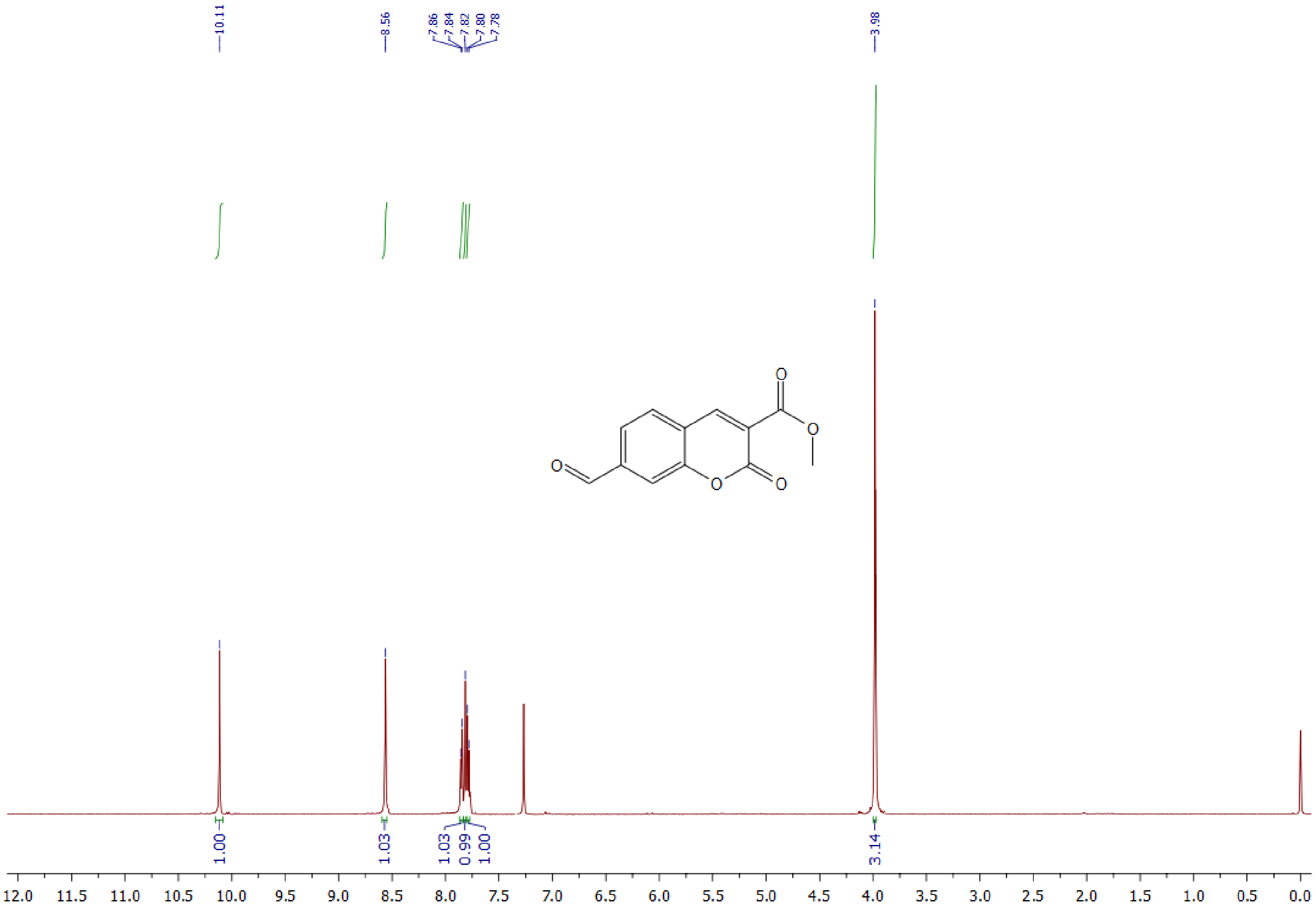

Fig. S8 500 MHz ^{1}H NMR spectrum of 2b in $CDCl_3$

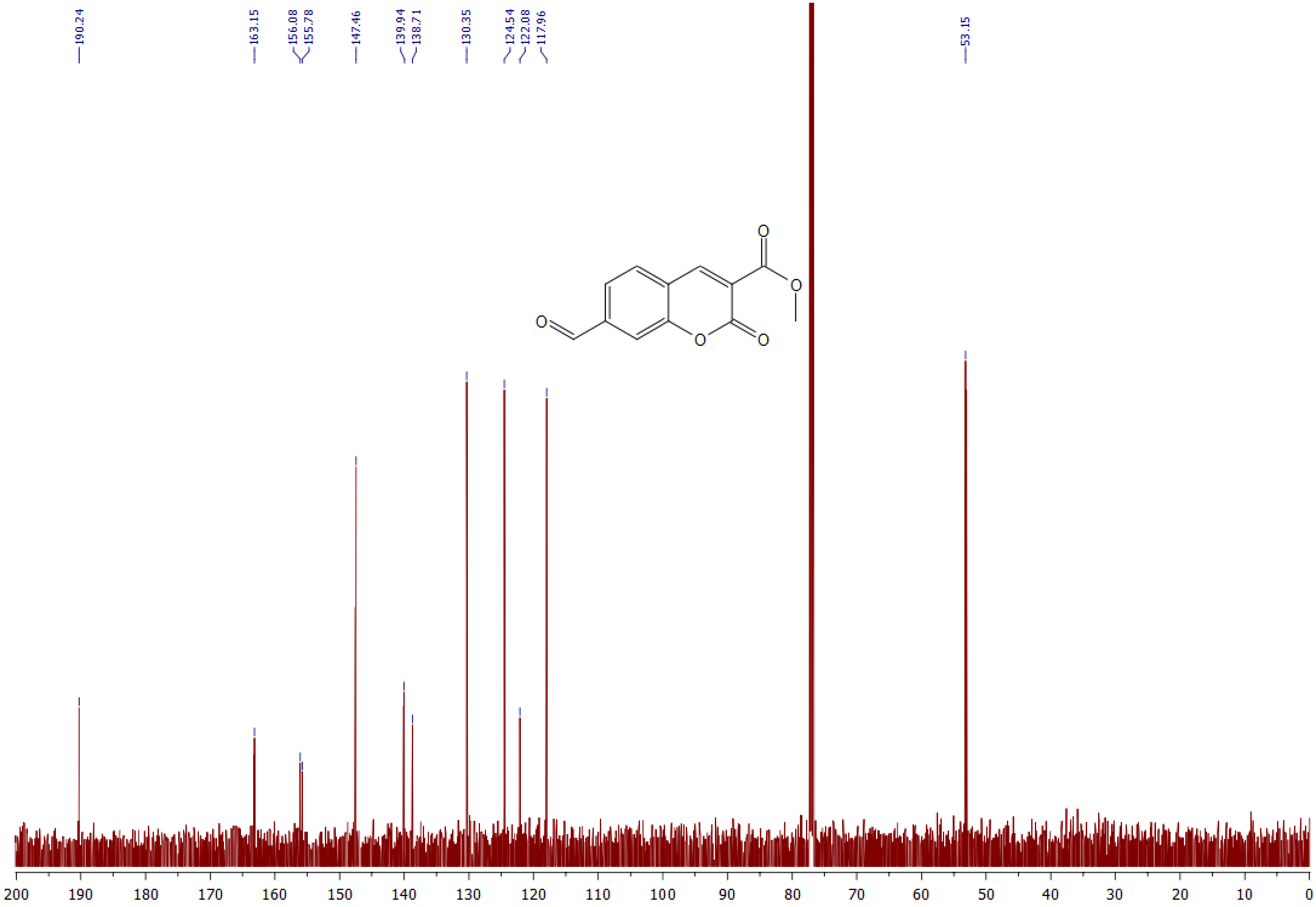

Fig. S9 500 MHz ^{13}C {^1H} NMR spectrum of 2b in $CDCl_3$

7. 500 MHz NMR spectra of Coum6 in $CDCl_3$

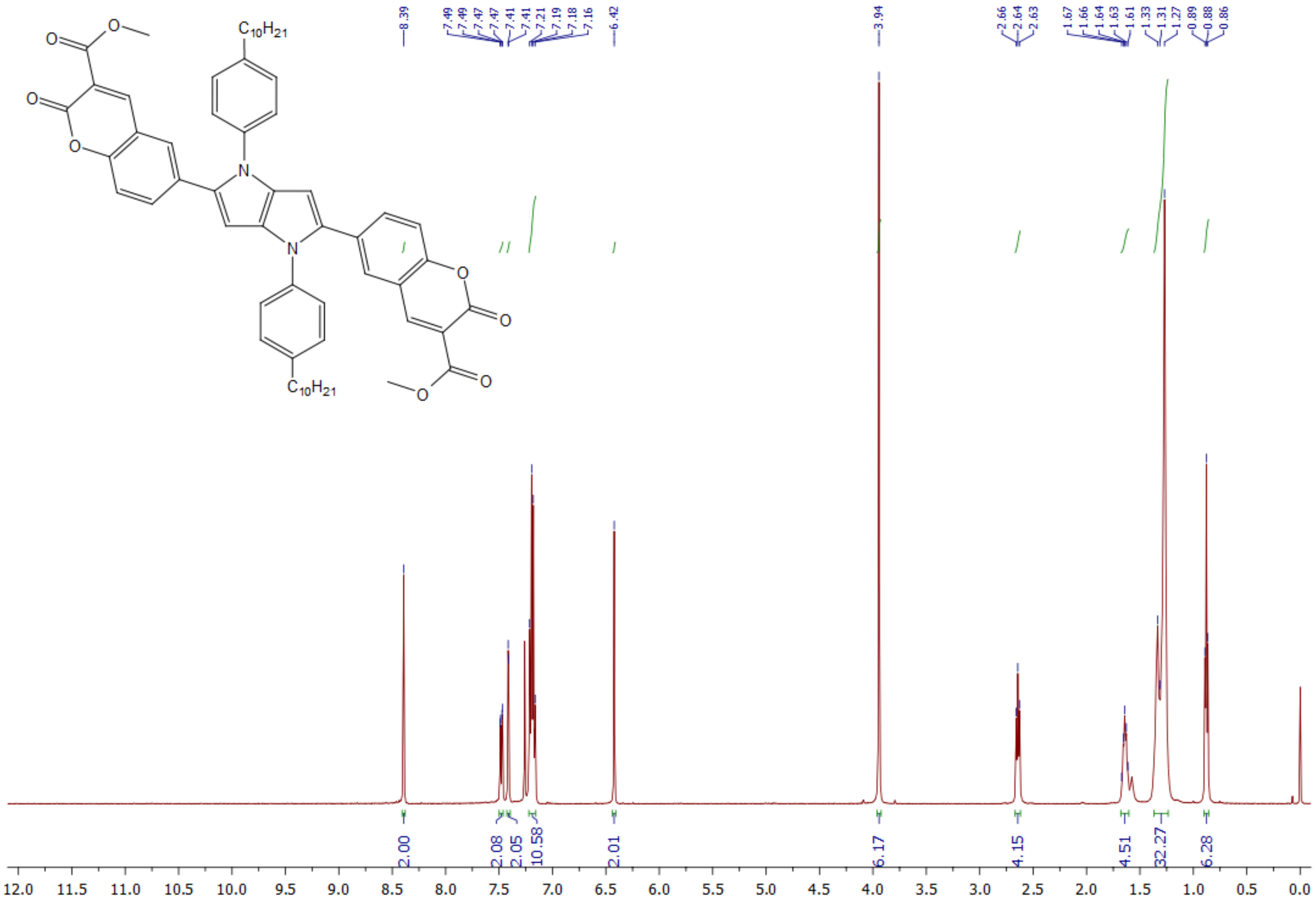

Fig. S10 500 MHz ^1H NMR spectrum of Coum6 in $CDCl_3$

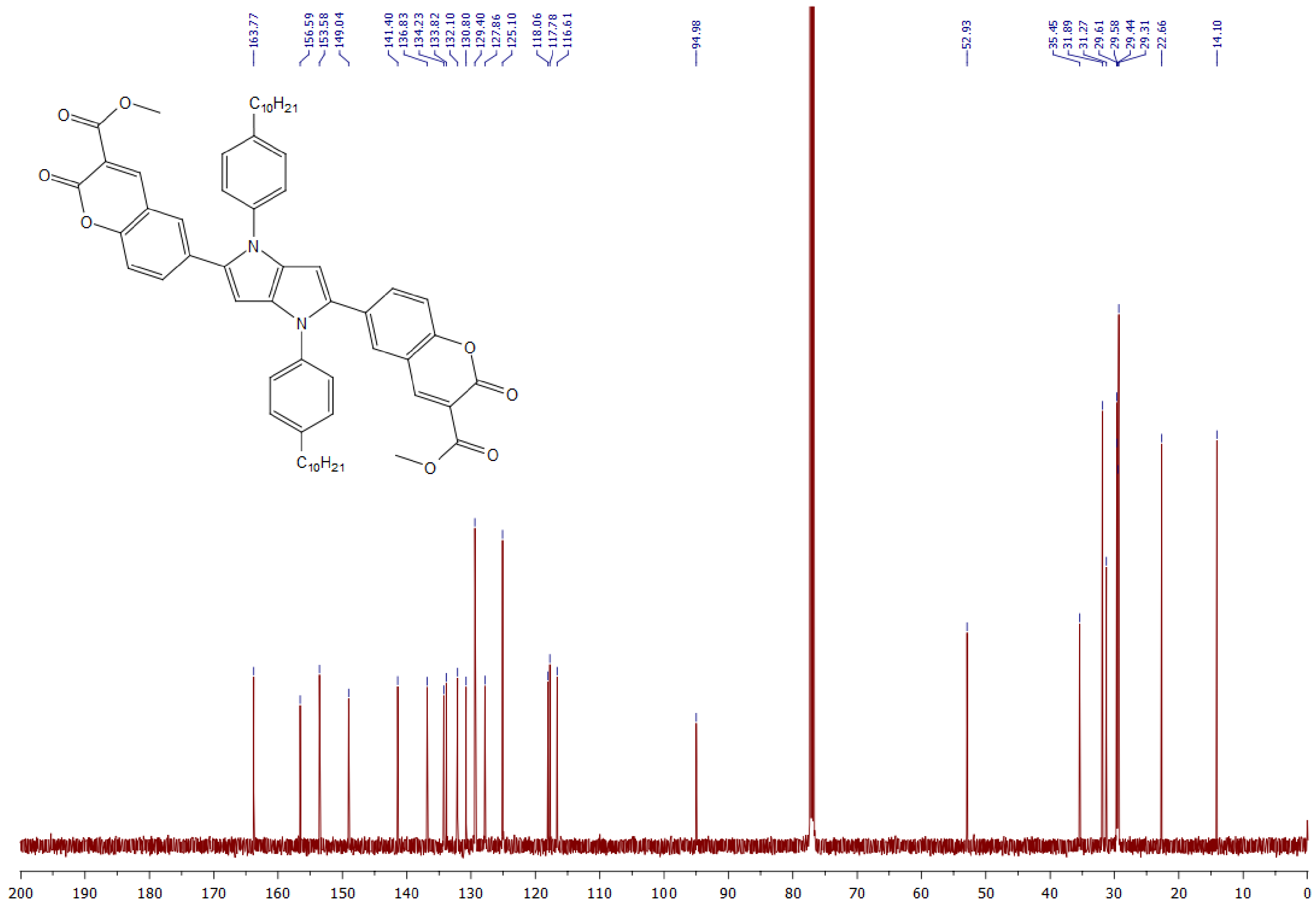

Fig. S11 500 MHz ^{13}C {^{1}H} NMR spectrum of Coum6 in $CDCl_3$

8. 500 MHz NMR spectra of Coum7 in $CDCl_3$

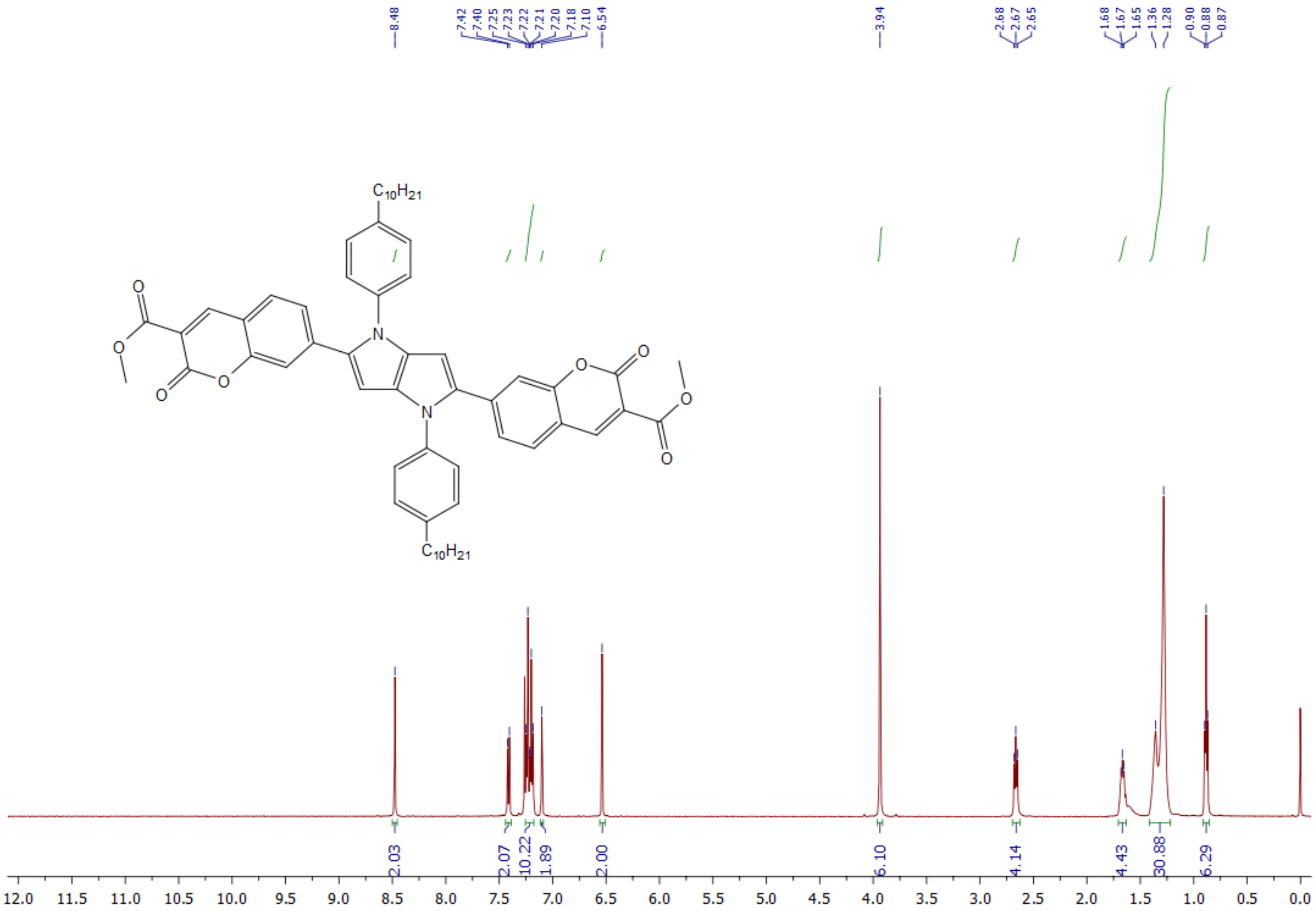

Fig. S12 500 MHz ^1H NMR spectrum of Coum7 in $CDCl_3$

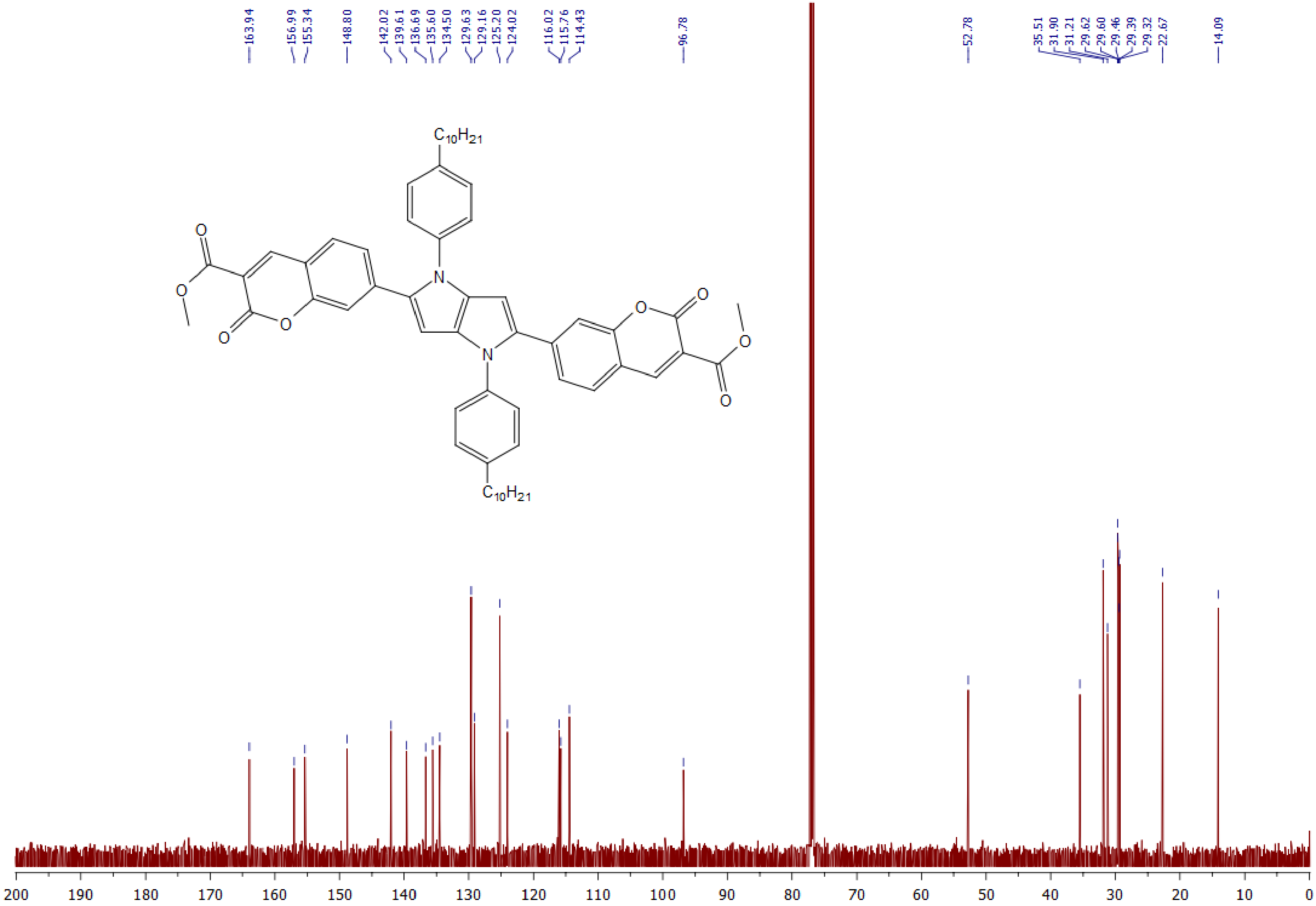

Fig. S13 500 MHz ^{13}C {^{1}H} NMR spectrum of Coum7 in $CDCl_3$

9. Two-photon absorption measurements of Coum6 and Coum7:

Two-photon absorption (TPA) spectrum of **Coum6** and **Coum7** are measured by the open-aperture Z-scan method[S6] with a femtosecond optical parametric amplifier (Spectra-Physics TOPAS Prime) as light source. The setup and the details of the analysis were reported previously.[S7] The measurements were performed by combining two different procedures: (1) scanning excitation wavelength for a fix incident power (wavelength scan) and (2) changing incident power to check the excitation intensity dependence (power scan). By wavelength scan, the spectral profile was quickly obtained and by power scan, it was confirmed that the observed non-linear absorption signal truly originates from TPA process for some important wavelengths. Some selected open-aperture Z-scan traces are shown in Figs. S14-S16 as examples. The z-scan traces were curve fitted with the theoretical equation which describe the transmittance of spatially and temporally Gaussian pulsed beam (eq. 1) to extract on-axis two-photon absorbance of the sample solution at the focal point.

$$T(\zeta) = T_N \frac{1}{\sqrt{\pi}\, q(\zeta)} \int_{-\infty}^{+\infty} \ln\left[1 + q(\zeta)\, e^{-x^2}\right] dx \qquad (1)$$

Where T_N is linear transmittance of the sample. ζ is normalized sample position $\zeta = z/z_R$ with the Rayleigh length z_R. $q(\zeta)$ is on-axis two-photon absorbance $q(\zeta) = q_0/(1+\zeta^2)$ with the two-photon absorbance at the sample position $q(0) = q_0$. From the change of q_0 against the incident power, i.e., excitation intensity, two-photon absorption coefficient β of the sample was obtained. TPA cross section $\sigma^{(2)}$ was finally obtained from β by using the convention $\sigma^{(2)} = h\nu\beta/N$, where $h\nu$ is photon energy of excitation pulse and N is number density of sample molecule calculated from the concentration. **Coum6** and **Coum7** were dissolved in 2-methylthterahydrofuran (MeTHF, spectroscopic grade from Aldrich). For **Coum6**, the concentration was 1.3 mM, close to the saturation concentration at room temperature. Solubility of **Coum7** was worse, so the solution was heated to around 50°C to keep **Coum7** dissolved at 0.5 mM. Solution was hold in 2-mm cuvette whose path length is short enough against z_R (4-6 mm) to satisfy the thin sample condition for eq. 1. No significant degradation was observed for both samples after Z-scan measurements. Inhouse standard materials were measured with the samples (GaN for <600 nm; MPPBT in dimethyl sulfoxide[S8] for 600–950 nm; rhodamine B in methanol[S9] for >950 nm) .

[S6] M. Sheik-Bahae, A. A. Said, T. H. Wei, D. J. Hagan, E. W. van Stryland, *IEEE J. Quantum Electron.,* **1990**, *26*, 760–769.

[S7] Kamada, K.; Matsunaga, K.; Yoshino, A.; Ohta, K.; *J. Opt. Soc. Am. B,* **2003**, *20*, 529–537.

[S8] K. Kamada, Y. Iwase, K. Sakai, K. Kondo, K. Ohta, *J. Phys. Chem. C*, **2009**, *113*, 11469-11474.

[S9] N. S. Makarov, M. Drobizhev and A. Rebane, *Opt. Express*, **2008**, *16*, 4029-4047.

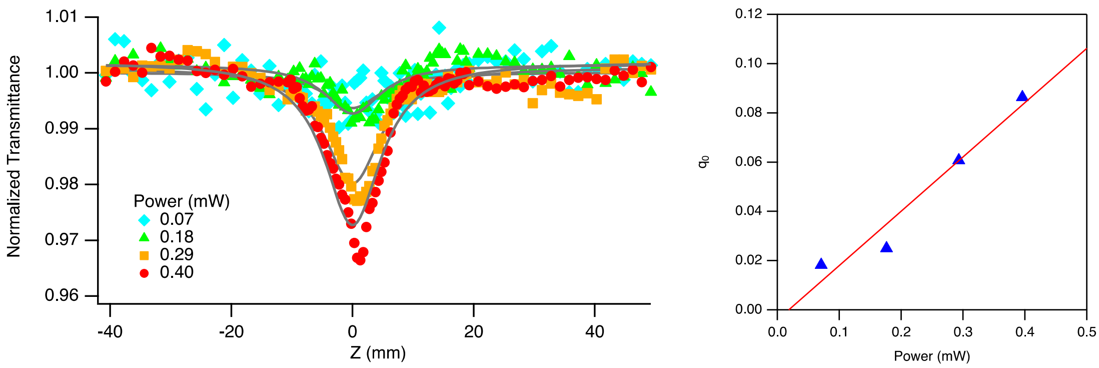

Fig. S14 (left) Open-aperture Z-scan traces of **Coum6** in MeTHF (1.3 mM) measured at 571 nm at different incident powers (symbols) with theoretical fits (grey curves). (right) The corresponding plot of the two-photon absorbance q_0 obtained from the curve fits against the incident power.

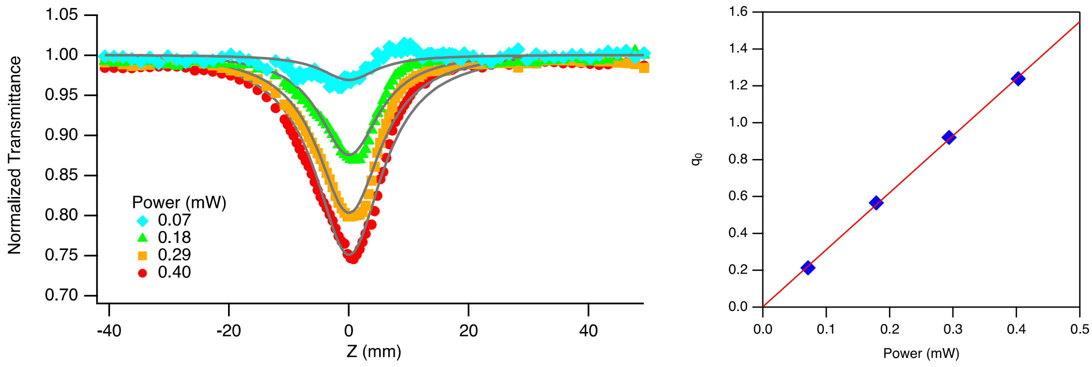

Fig. S15 (left) Open-aperture Z-scan traces of **Coum7** in MeTHF (0.5 mM) measured at 571 nm at different incident powers (symbols) with theoretical fits (grey curves). (right) The corresponding plot of the two-photon absorbance q_0 obtained from the curve fits against the incident power.

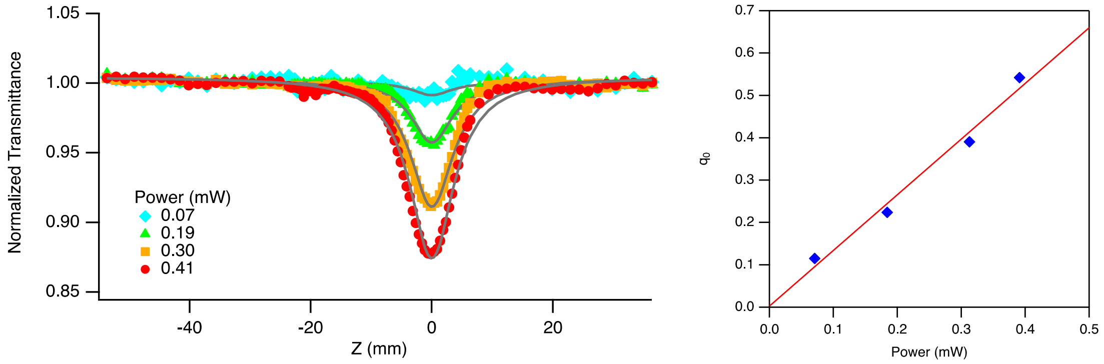

Fig. S16 (left) Open-aperture Z-scan traces of **Coum7** in MeTHF (0.5 mM) measured at 845 nm at different incident powers (symbols) with theoretical fits (grey curves). (right) The corresponding plot of the two-photon absorbance q_0 obtained from the curve fits against the incident power.

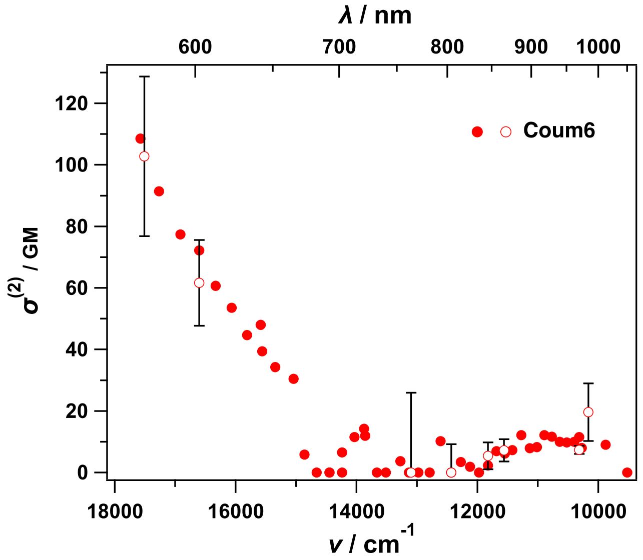

Fig. S17 Spectrum of two-photon absorption cross section ($\sigma^{(2)}$) of **Coum6** in MeTHF (1.3 mM) against the excitation photon energy in cm^{-1} (ν) in bottom and the corresponding wavelength (λ) in top. The filled circles mean the data measured at a fixed incident power (0.32 or 0.40 mW, wavelength sacan) and the open circles with error bars are the results of the excitation power dependence of at least four different excitation powers (power scan).
